# Mycobacterium tuberculosis MmsA (Rv0753c) Interacts with STING and Blunts the Type I Interferon Response

**DOI:** 10.1128/mBio.03254-19

**Published:** 2020-12-01

**Authors:** Yifan Sun, Wei Zhang, Chunsheng Dong, Sidong Xiong

**Affiliations:** a Jiangsu Key Laboratory of Infection and Immunity, The Institutes of Biology and Medical Sciences, Soochow University, Suzhou, Jiangsu, China; Max Planck Institute for Infection Biology

**Keywords:** STING, *Mycobacterium tuberculosis*, type I IFN, MmsA (Rv0753c), p62, selective autophagy

## Abstract

Type I interferon (IFN) plays an important role in Mycobacterium tuberculosis persistence and disease pathogenesis. M. tuberculosis has evolved a number of mechanisms to evade host immune surveillance. However, it is unclear how the type I IFN response is tightly regulated by the M. tuberculosis determinants. Stimulator of interferon genes (STING) is an essential adaptor for type I IFN production triggered by M. tuberculosis genomic DNA or cyclic dinucleotides upon infection. To investigate how the type I IFN response is regulated by M. tuberculosis determinants, immunoprecipitation-mass spectrometry-based (IP-MS) proteomic analysis was performed to screen proteins interacting with STING in the context of M. tuberculosis infection. Among the many predicted candidates interacting with STING, the M. tuberculosis coding protein Rv0753c (MmsA) was identified. We confirmed that MmsA binds and colocalizes with STING, and the N-terminal regions of MmsA (amino acids [aa] 1 to 251) and STING (aa 1 TO 190) are responsible for MmsA-STING interaction. Type I IFN production was impaired with exogenous expression of MmsA in RAW264.7 cells. MmsA inhibited the STING-TBK1-IRF3 pathway, as evidenced by reduced STING levelS and subsequent IRF3 activation. Furthermore, MmsA facilitated p62-mediated STING autophagic degradation by binding p62 with its C terminus (aa 252 to 455), which may account for the negative regulation of M. tuberculosis MmsA in STING-mediated type I IFN production. Additionally, the M. tuberculosis *mmsA* R138W mutation, detected in a hypervirulent clinical isolate, enhanced the degradation of STING, implying the important relevance of MmsA in disease outcome. Together, we report a novel mechanism where M. tuberculosis MmsA serves as an antagonist of type I IFN response by targeting STING with p62-mediated autophagic degradation.

## Abstract (precis)

It is unclear how the type I IFN response is regulated by mycobacterial determinants. Here, we characterized the previously unreported role of M. tuberculosis MmsA in immunological regulation of type I IFN response by targeting the central adaptor STING in the DNA sensing pathway. We identified STING-interacting MmsA by coimmunoprecipitation-mass spectrometry-based (IP-MS) proteomic analysis and showed MmsA interacting with STING and autophagy receptor p62 via its N terminus and C terminus, respectively. We also showed that MmsA downregulated type I IFN by promoting p62-mediated STING degradation. Moreover, the MmsA mutant R138W is potentially associated with the virulence of M. tuberculosis clinical strains owing to the modulation of STING protein. Our results provide novel insights into the regulatory mechanism of type I IFN response manipulated by mycobacterial MmsA and the additional cross talk between autophagy and STING in M. tuberculosis infection, wherein a protein from microbial pathogens induces autophagic degradation of host innate immune molecules.

## INTRODUCTION

Tuberculosis, caused by Mycobacterium tuberculosis, is a fatal chronic infectious disease. According to the latest global tuberculosis report, this disease affected 10 million individuals in 2018, and it is estimated that approximately 1.7 billion individuals have a latent tuberculosis infection (LTBI) (1). Although the type I interferon (IFN) response is essential for host defense against viral infections, its role in bacterial infections remains controversial (2–4). The pathological role of type I IFN in tuberculosis was first reported through transcriptomic analysis, and this study confirmed that type I IFN is a key biomarker of active tuberculosis and is associated with the severity of the disease (5). A hypervirulent clinical isolate, W4 (W/Beijing strain), significantly induces increased IFN-α mRNA synthesis compared to that with CDC1551 (non-W/Beijing strain) (6). Further evidence indicated that the infection of a clinically virulent M. tuberculosis isolate associated with the induction of type I IFN results in the impairment of protective Th1 immunity (7). There are also some reports showing that IFN treatment during chronic viral infections increases the susceptibility to tuberculosis (2, 4, 8, 9). However, other studies reported type I IFN also acts as a potentially protective cytokine in tuberculosis models. In contrast to what has been reported in the immunocompetent host, where type I IFN signaling may promote local accumulation of permissive myeloid cells that contribute to the spread of infection and pulmonary inflammation, it may play a nonredundant protective role in the absence of IFN-γ signaling and is critical for the optimal recruitment of myeloid cells in the lungs to restrict the bacterial burdens and histopathology in M. tuberculosis infection (10). Therapeutic effects of IFN-α have also been reported in young patients suffering from mycobacterial infections with complete or partial IFN-γ receptor signaling deficiencies when administered together with antimycobacterial chemotherapy (11, 12).

Stimulator of interferon genes (STING; also known as ERIS, MITA, or MPYS) is an important adaptor molecule involved in innate immunity, linking cytosolic DNA sensing and cell activation. It also senses cyclic dinucleotides by itself, leading to the induction of type I IFN (13–15). STING plays central roles in type I IFN induction upon M. tuberculosis infection (16–20). In 2012, Manzanillo et al. first reported that STING is an essential component of IRF3 activation and IFN-β transcription during M. tuberculosis infection (16). Further studies have reported that both M. tuberculosis genomic DNA and the host mitochondrial DNA bind to the primary DNA sensor cyclic GMP-AMP synthase (cGAS) to activate the STING-TBK1-IRF3 pathway in response to different M. tuberculosis strains, such as Erdman and CDC1551 (17–20). Mycobacterial cyclic dinucleotide c-di-AMP, a key pathogen-associated molecular pattern, also triggers STING signaling in host cells, inducing type I IFN responses (21). Accordingly, a recombinant BCG (BCG-disA-OE) vaccine expressing M. tuberculosis deadenylate cyclase for a high level of STING agonist c-di-AMP enhances the protective effect against M. tuberculosis infection (22). It has also been reported that during M. tuberculosis infection, STING is closely related with autophagy. Watson et al. first revealed that direct activation of STING is necessary for the formation of autophagosome targeting to M. tuberculosis (23). Subsequent studies further provided evidence on how the essential adaptor protein STING affects the selective autophagy pathway (17, 19).

Although the differential accumulation of host mitochondrial DNA may account for the increase in STING-dependent IFN-β induction in the phylogenetic lineages of M. tuberculosis (20), other bacterial determinants could also be involved in this regulatory mechanism. For M. tuberculosis, Rv2837c, also known as CdnP, inhibits the cGAS-cGAMP-STING pathway through the degradation of both bacterially derived canonical cyclic dinucleotides (CDNs) and host-derived noncanonical CDNs to evade the immune system (24). Another study reported that the interaction between activated apoptosis-associated speck-like protein containing a caspase recruitment domain (ASC) and STING impedes STING-TBK1 interaction and type I IFN induction, protecting the host from M. tuberculosis infection. Although both studies reported negative regulation of the STING pathway, the aforementioned findings appear contradictory, suggesting that the modulation of STING signaling in M. tuberculosis infection is more complex. Therefore, further studies are required to determine how STING is tightly regulated and affects downstream type I IFN induction and the outcome of M. tuberculosis infection.

The *Rv0753c* (*mmsA*) gene was first reported in the study of whole-genome sequencing of M. tuberculosis virulence strain H37Rv (25). It was predicted as a malonate-semialdehyde dehydrogenase and methylmalonate-semialdehyde dehydrogenase and is a secreted protein existing in the supernatant of M. tuberculosis culture filtrate (26). A previous study reported M. tuberculosis *mmsA* was upregulated in 28-day and 50-day cultures but not in 14-day cultures (27). The *mmsA* homologs belong to the coenzyme A (CoA)-dependent aldehyde dehydrogenase (Aldedh) subfamily, catalyzing the NAD-dependent oxidation of methylmalonate semialdehyde and malonate semialdehyde into propionyl-CoA and acetyl-CoA in a wide variety of organisms, ranging from bacteria to mammals. The MmsA from bacteria species such as Streptomyces coelicolor is involved in valine catabolism (28). In mammals, MmsA is a mitochondrial enzyme that participates in the distal portions of the valine and pyrimidine catabolic pathways (29). M. tuberculosis MmsA contains 510 amino acids with an Aldedh domain for enzymatic function according to UniProt analysis, while other functional domains of M. tuberculosis MmsA have not been reported yet. Although few studies report the M. tuberculosis MmsA dehydrogenase function, some studies showed that MmsA activates dendritic cells (DCs) and promotes the Th1 immune response during M. tuberculosis infection (30), and the diagnostic value of MmsA for LTBI screening from active tuberculosis has also been emphasized (31–34). However, the mechanism underlying MmsA-mediated regulation of the host innate immune response is unclear. This study aimed to provide novel insights into the mechanism underlying the interaction between M. tuberculosis MmsA and host STING and the regulation of the type I IFN response. Coimmunoprecipitation (co-IP) and mass spectrometry were performed to identify the mycobacterial STING-interacting proteins, revealing MmsA as a promising candidate to interact with STING and blunt the type I IFN response via p62-mediated autophagic degradation.

## RESULTS

### M. tuberculosis MmsA is a binding partner of STING.

**FIG 1:**
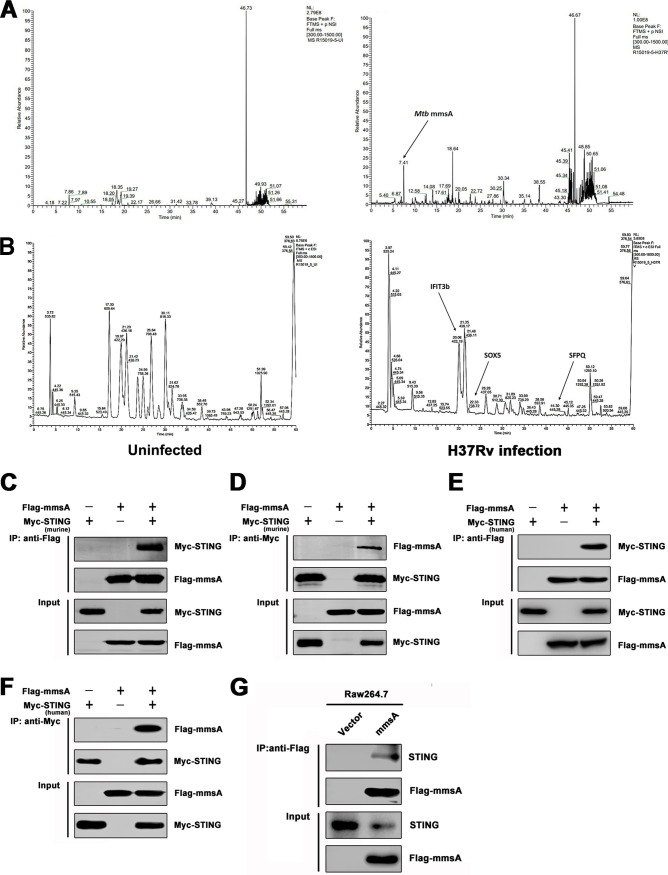
M. tuberculosis MmsA is a binding partner of STING. RAW264.7 cells were infected with H37Rv for 6 h at an MOI of 10, and then the cell lysates were subjected to immunoprecipitation with anti-STING antibody or normal IgG. The immunoprecipitates were subjected to SDS-PAGE and identified by mass spectroscopy, which showed the mass spectroscopy spectrum of the STING protein complex searched against the mycobacterial proteome (A) and the mouse proteome (B). The arrows indicate the peaks of the candidate proteins interacting with STING as mycobacterial MmsA (A) and as murine IFIT3b, SOX5, and SFPQ (B) in H37Rv-infected (right) cells. HEK293T cells were transfected with vector or Flag-tag MmsA and murine Myc-tag STING for 48 h. Cell lysates were immunoprecipitated with the anti-Flag antibody (C) or anti-Myc antibody (D) and then immunoblotted with the indicated antibodies. HEK293T cells were transfected with vector or Flag-tag MmsA and human Myc-tag STING for 48 h. Cell lysates were immunoprecipitated with the anti-Flag antibody (E) or anti-Myc antibody (F) and then immunoblotted with the indicated antibodies. (G) The cell lysates from RAW264.7 cells stably expressing Flag-MmsA (RAW-MmsA) and RAW-Vector were immunoprecipitated with the anti-Flag antibody and analyzed by immunoblotting with the indicated antibodies.

STING is an essential adaptor for the production of type I IFNs triggered by M. tuberculosis genomic DNA and cyclic dinucleotide c-di-AMP (16–19, 21). However, the regulation of STING-mediated type I IFN signaling during M. tuberculosis infection remains largely unknown. To identify new factors modulating this pathway, the murine macrophage cell line RAW264.7 was infected with the M. tuberculosis strain H37Rv, and the STING protein complex was analyzed by co-IP and mass spectrometry. The arrows indicate the peaks of the candidate proteins from mycobacteria (Fig. 1A) or mice (Fig. 1B) that interacted with STING. Bioinformatics analysis indicated that some host proteins, such as SOX5, SFPQ, and IFIT3b, were coimmunoprecipitated with STING; of these, SFPQ was previously suggested to be a binding partner of STING (35). Notably, the co-IP and subsequent analysis revealed that M. tuberculosis protein MmsA (Rv0753c) also had a high binding score (see Table S1 in the supplemental material). Because we were interested in elucidating how mycobacterial proteins modulate the type I IFN response, we focused on MmsA. To confirm the STING-MmsA binding observed by immunoprecipitation-mass spectrometry-based (IP-MS) in RAW264.7 cells, we further performed the co-IP of Myc-tagged murine STING and Flag-tagged MmsA in HEK293T cells. As expected, M. tuberculosis Flag-MmsA could bind Myc-STING (Fig. 1C). Reversible co-IP in HEK293T cells also demonstrated the binding between STING and MmsA (Fig. 1D). Moreover, exogenous co-IP indicated that MmsA associated not only with murine STING but also with human STING (Fig. 1E and F). In RAW264.7 cells stably expressing Flag-MmsA, MmsA was found to bind endogenous STING (Fig. 1G) but not the other critical innate immune proteins, cGAS and MAVS (Fig. S1A and B). In addition, we also cotransfected plasmids expressing MmsA homolog protein of Mycobacterium smegmatis (M. smegmatis MmsA) in HEK293T cells for co-IP experiments. Our finding showed that M. tuberculosis MmsA, but not M. smegmatis-MmsA, could bind STING, suggesting the binding specificity between STING and M. tuberculosis MmsA (Fig. S1C). Taken together, these data indicated that M. tuberculosis MmsA and STING associate with each other.

10.1128/mBio.03254-19.1 FIG S1 M. tuberculosis MmsA interacts with STING. HEK293T cells were transfected with pFlag-cGAS (A) or pFlag-MAVS (B), with or without pMyc-MmsA, for 48 h. Cell lysates were immunoprecipitated with the anti-Myc antibody, followed by immunoblotting with the indicated antibodies. (C) HEK293T cells were transfected with pFlag-MS-MmsA or pFlag-MmsA (M. tuberculosis) together with pMyc-human-STING for 48 h. Cell lysates were immunoprecipitated with the anti-Flag antibody, followed by immunoblotting with the indicated antibodies. Download FIG S1, TIF file, 0.6 MB. Copyright © 2020 Sun et al. 2020 Sun et al. This content is distributed under the terms of the Creative Commons Attribution 4.0 International license.

10.1128/mBio.03254-19.5 TABLE S1 Candidate STING interacting proteins identified by IP-MS approach. Download Table S1, DOCX file, 0.01 MB. Copyright © 2020 Sun et al. 2020 Sun et al. This content is distributed under the terms of the Creative Commons Attribution 4.0 International license.

### M. tuberculosis MmsA colocalizes with STING.

**FIG 2:**
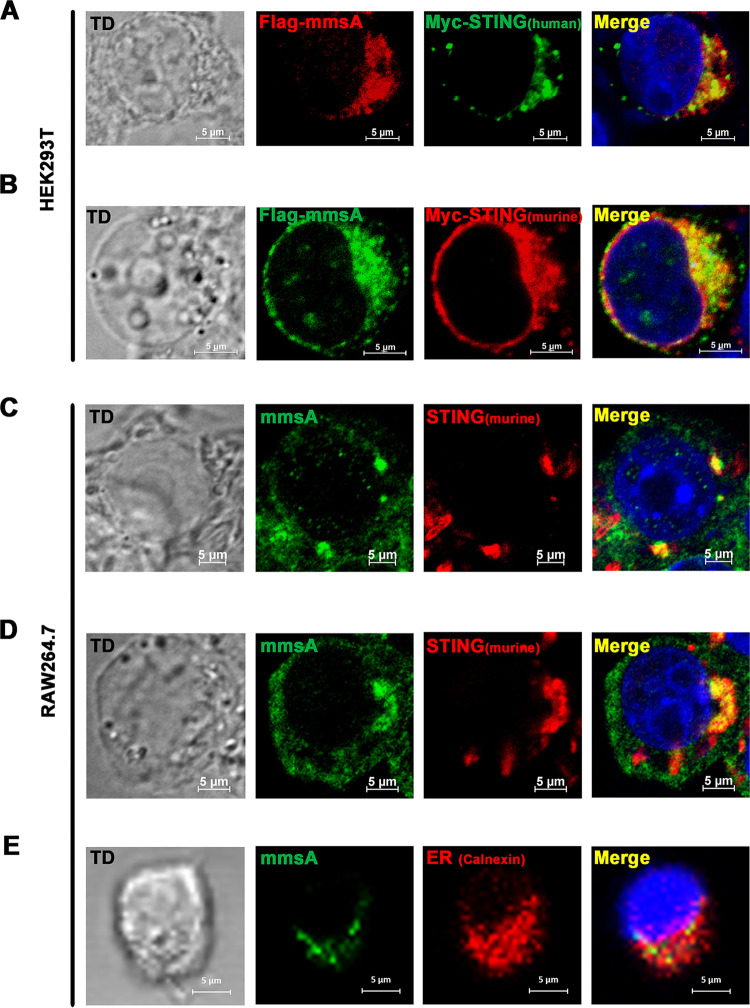
M. tuberculosis MmsA colocalizes with STING. HEK293T cells were transfected with pMyc-human-STING plasmid (A) or pMyc-murine-STING plasmid (B) along with equal amounts of pFlag-MmsA plasmid. After 36 h, the cells were processed for confocal microscopy with anti-Flag antibody (MmsA) and anti-Myc antibody (STING). RAW264.7 cells were infected with M. smegmatis::MmsA (C) or H37Rv (D). After 24 h, the cells were processed for confocal microscopy with anti-STING antibody (red) and anti-MmsA antibody (green). (E) RAW264.7 cells were infected with M. smegmatis::MmsA for 12 h. The ER marker calnexin (green) and MmsA (red) were stained in the infected cells, and the cells were observed under confocal microscopy. Nuclei were stained with DAPI. Scale bars, 5 μm. TD, transmitted light channel.

STING was reported to localize predominantly in the ER (13, 36), and pathogen proteins targeting STING, such as HCV NS4B, also localized predominantly in the endoplasmic reticulum (ER) (37). To determine the colocalization of MmsA and STING, we cotransfected Flag-MmsA and Myc-tagged STING into HEK293T cells. Confocal microscope images showed that MmsA and human STING (Fig. 2A), as well as murine STING (Fig. 2B), were mainly located in the cytoplasm of transfected cells and colocalized well with each other. A nonpathogenic species, Mycobacterium smegmatis, has been widely used as a model for mycobacterial functional studies due to its fast growth and safety (38). Therefore, we generated the recombinant M. smegmatis expressing M. tuberculosis MmsA (M. smegmatis::MmsA). To examine MmsA-STING colocalization in the context of mycobacterial infection, we infected the RAW264.7 cells with M. smegmatis::MmsA. Immunofluorescence assay showed that the endogenous MmsA expressed by M. smegmatis::MmsA indeed colocalized with STING (Fig. 2C). Similar results were obtained from MmsA expressed via infection with H37Rv in RAW264.7 cells (Fig. 2D). Moreover, we found that MmsA in M. smegmatis::MmsA-infected RAW264.7 cells colocalized with the ER marker calnexin (Fig. 2E). These results suggested that M. tuberculosis MmsA colocalizes with STING.

### The N-terminal region of STING (aa 1 to 190) and MmsA (aa 1 to 251) is crucial for their interaction.

**FIG 3:**
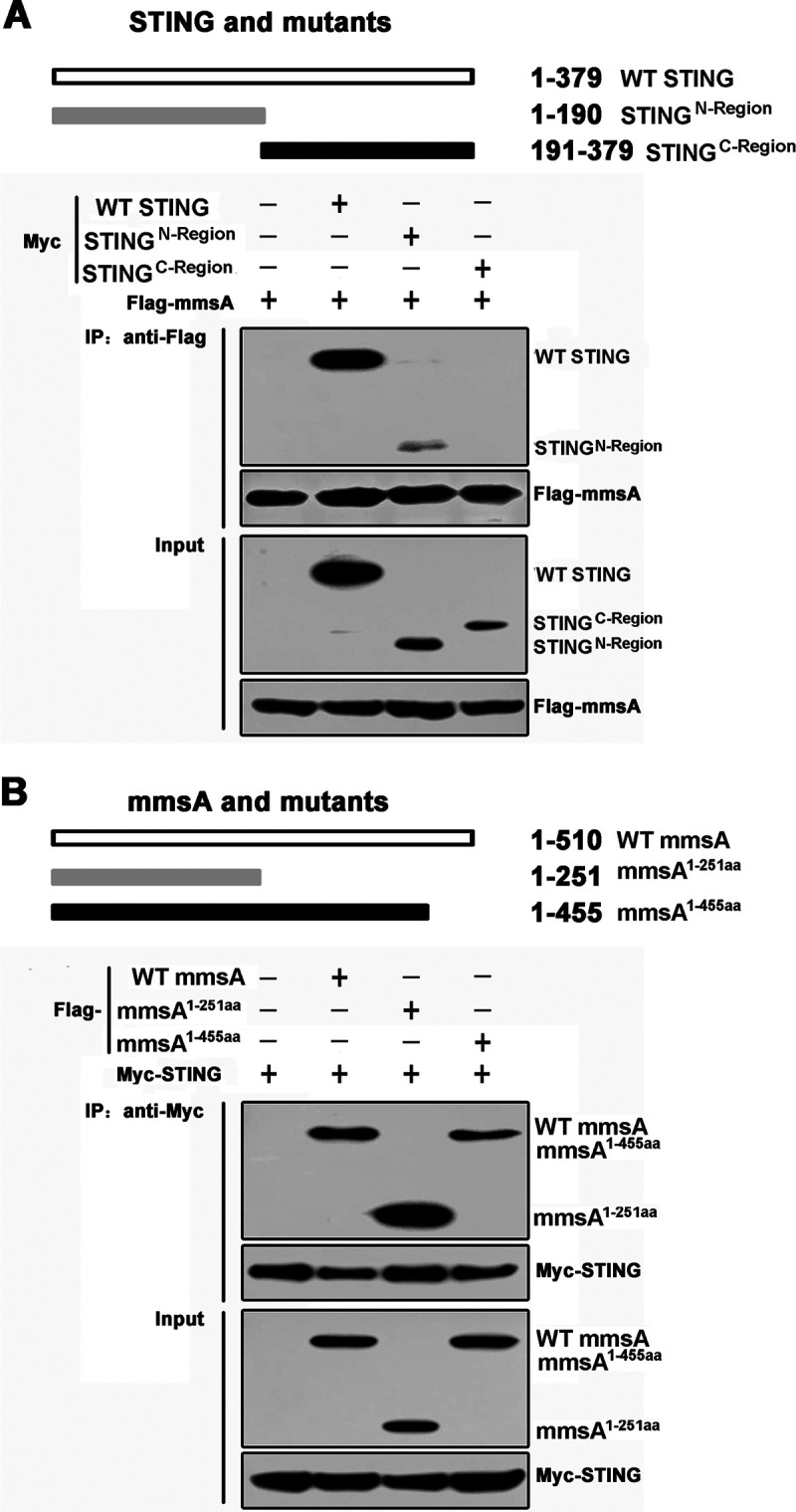
Interaction of MmsA and STING depends on the N-terminal region of both proteins. (A) Schematic diagram of STING and its truncation mutants. pMyc-human-STING or its mutants pSTING^N-Region^ and pSTING^C-Region^ were individually transfected into HEK293T cells along with pFlag-MmsA. Cell lysates were immunoprecipitated with the anti-Flag antibody and then immunoblotted with the indicated antibodies. (B) Schematic diagram of MmsA and its truncation mutants. pFlag-MmsA or its mutants, pFlag-MmsA^1-251aa^ and pFlag-MmsA^1-451aa^, were individually transfected into HEK293T cells along with pMyc-human-STING. Cell lysates were immunoprecipitated with the anti-Myc antibody and then immunoblotted with the indicated antibodies.

The human STING is a 379-amino-acid protein. The amino-terminal domain (aa 1 to 152) of STING contains four transmembrane domains for the cellular location, such as endoplasmic reticulum and mitochondrion. A central globular domain contains a dimerization region (aa 153 to 177) and a cyclic dinucleotide binding domain (aa 178 to 341). The C-terminal tail (aa 342 to 379) is required for downstream signaling (39–41). To investigate the regions necessary for the MmsA and STING interaction, we first truncated Myc-tagged STING as an N-terminal (aa 1 to 190) truncation mutant, STING^N-Region^, or C-terminal (aa 191 to 379) truncation mutant, STING^C-Region^ (42, 43). Interestingly, in HEK293T cells we found that the N-terminal region of STING, but not the C-terminal region, was critical for the interaction between STING and MmsA (Fig. 3A). We then generated different truncation mutations of MmsA, i.e., MmsA^1–455aa^ and MmsA^1–251aa^, based on the domain architecture of MmsA homologs from Sinorhizobium meliloti and Bacillus subtilis. We found that both MmsA^1-455aa^ and MmsA^1-251aa^ could similarly mediate the interaction of MmsA with STING (Fig. 3B). Thus, we mapped the N-terminal region of MmsA (1 to 251 aa) as essential for the interaction between MmsA and STING. Collectively, these results indicated that the N-terminal protein regions of both MmsA and STING are crucial for their interaction.

### Binding of MmsA to STING inhibits type I IFN production via the STING-TBK1-IRF3 pathway.

**FIG 4:**
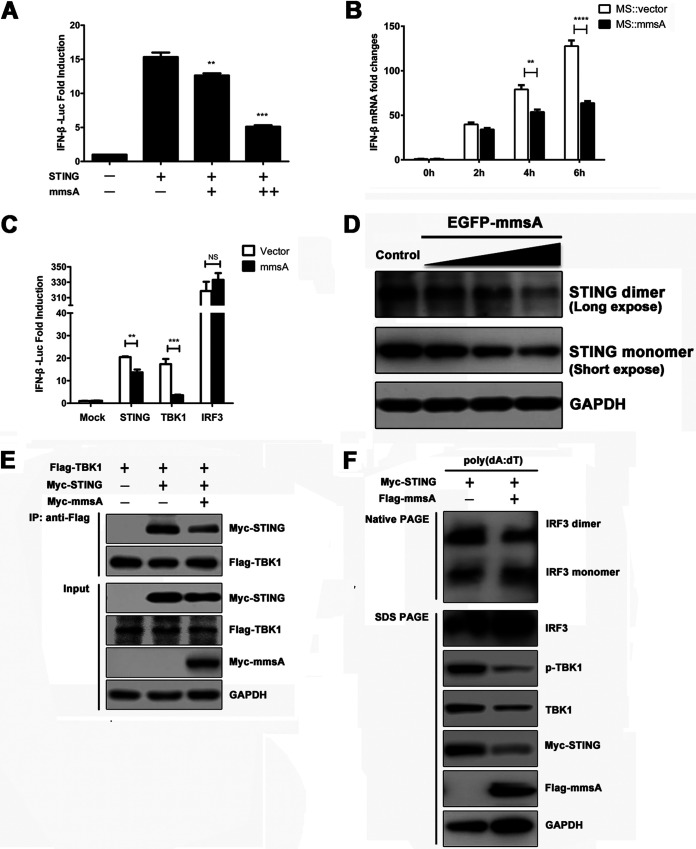
Binding of MmsA to STING inhibits type I IFN production via STING-TBK1-IRF3 pathway. (A) HEK293T cells were cotransfected with the interferon reporter plasmid IFN-β-Luc together with the plasmids expressing human STING and increasing amounts of M. tuberculosis MmsA, with a *Renilla* luciferase plasmid as an internal control. Luciferase activities were determined 24 h posttransfection. (B) RAW264.7 cells were infected with M. smegmatis::vector and M. smegmatis::MmsA for 0, 2, 4, and 6 h at an MOI of 10, and IFN-β mRNA was measured by qPCR. GAPDH was used as a loading control. (C) HEK293T cells were cotransfected with the interferon reporter plasmid IFN-β-Luc together with the plasmids expressing M. tuberculosis MmsA and STING, TBK1, or IRF3. A *Renilla* luciferase plasmid was an internal control. Luciferase activities were determined 24 h posttransfection. (D) RAW264.7 cells were transfected with empty vector or the plasmids expressing EGFP-MmsA in increasing amounts of 0.5 μg, 1.0 μg, and 2.0 μg. Forty-eight hours posttransfection, STING monomer and dimer were analyzed by immunoblotting. (E) HEK293T cells were transfected with pMyc-human-STING and Flag-TBK1, with or without pMyc-MmsA, for 48 h. Cell lysates were immunoprecipitated with the anti-Flag antibody and then immunoblotted with the indicated antibodies. (F) Raw264.7 cells were transfected with plasmid pMyc-murine-STING and vector or pFlag-MmsA for 20 h and treated with poly(dA:dT) (2 μg/ml) for 8 h. Cell lysates were analyzed for IRF3 dimerization by native PAGE. Total IRF3 expression levels and other indicated proteins were analyzed by immunoblotting. The results are shown as means ± SEM. NS, not significant; **, *P < *0.01; ***, *P < *0.001; ****, *P < *0.0001; each by Student's *t* test.

STING is a key molecule for the cytosolic DNA- and cyclic dinucleotide-induced production of type I IFN. To detect the effect of MmsA-STING binding on type I IFN production and signaling, we performed an IFN-β reporter assay in MmsA-overexpressing HEK293T cells. IFN-β activation was reduced upon the exogenous expression of MmsA in a dose-dependent manner (Fig. 4A). To further confirm the inhibitory effect of MmsA on IFN-β induction, the transcription of IFN-β was measured by qPCR upon mycobacterial infection. Consistent with the IFN-β reporter assay, MmsA also downregulated type I IFN production in M. smegmatis::MmsA-infected RAW264.7 cells compared to that in the M. smegmatis::Vector-infected cells (Fig. 4B). Similar results were also obtained in poly(dA:dT)-stimulated RAW264.7 cells exogenously expressing M. tuberculosis MmsA (RAW-MmsA) and control RAW-Vector cells (Fig. S2A), demonstrating a novel role of MmsA in inhibiting type I IFN production.

10.1128/mBio.03254-19.2 FIG S2 Binding of MmsA to STING inhibits type I IFN production via STING-TBK1-IRF3 pathway. (A) RAW-MmsA and RAW-Vector cells were treated with poly(dA:dT) (2 μg/ml) for the indicated time, and IFN-β production was measured by qPCR. GAPDH used as a loading control. (B) HEK293T cells were transfected with plasmids expressing pMyc-human-STING and vector or pFlag-MmsA for 48 h. Cells were lysed and then immunoblotted with the indicated antibodies. The results are shown as means ± SEM. **, *P < *0.01; ***, *P < *0.001; each by Student *t* test. Download FIG S2, TIF file, 0.2 MB. Copyright © 2020 Sun et al. 2020 Sun et al. This content is distributed under the terms of the Creative Commons Attribution 4.0 International license.

To examine which of the steps in type I IFN production are affected by MmsA, we transfected plasmids expressing MmsA and key molecules involved in STING signaling, such as STING, TBK1, and IRF3, together with an IFN-β luciferase reporter plasmid, into HEK293T cells. The inhibition of IFN by MmsA was observed in STING- and TBK1-overexpressing cells. However, the production was no longer inhibited by the overexpression of constitutively active IRF3/5D, suggesting that MmsA suppresses the STING-mediated IFN signaling pathway upstream of IRF3 (Fig. 4C).

The STING-TBK1-IRF3 pathway is critical for type I IFN production, and activated STING recruits the kinase TBK1 to stimulate the phosphorylation of IRF3 (44); MmsA might also impair the activity of the STING-TBK1-IRF3 complex. To assess the effect of MmsA on the STING-TBK1-IRF3 axis, RAW264.7 cells were transfected with increasing amounts of plasmids expressing EGFP-MmsA, and we found that MmsA overexpression reduced STING dimerization, which might be due to the decreased level of total STING (Fig. 4D). Meanwhile, we also observed the reduction of STING dimerization in HEK293T cells cotransfected with STING- and MmsA-expressing plasmids (Fig. S2B). Consistent with this, reduced association between STING and TBK1 in MmsA-overexpressing HEK293T cells was observed (Fig. 4E). MmsA also antagonized poly(dA:dT)-triggered phosphorylation of TBK1 and dimerization of IRF3 in RAW263.4 cells (Fig. 4F). Taken together, these data indicated that MmsA targets the STING-TBK1-IRF3 pathway to repress the production of type I IFN upon DNA stimulation.

### MmsA mediates autophagy-dependent degradation of STING.

**FIG 5:**
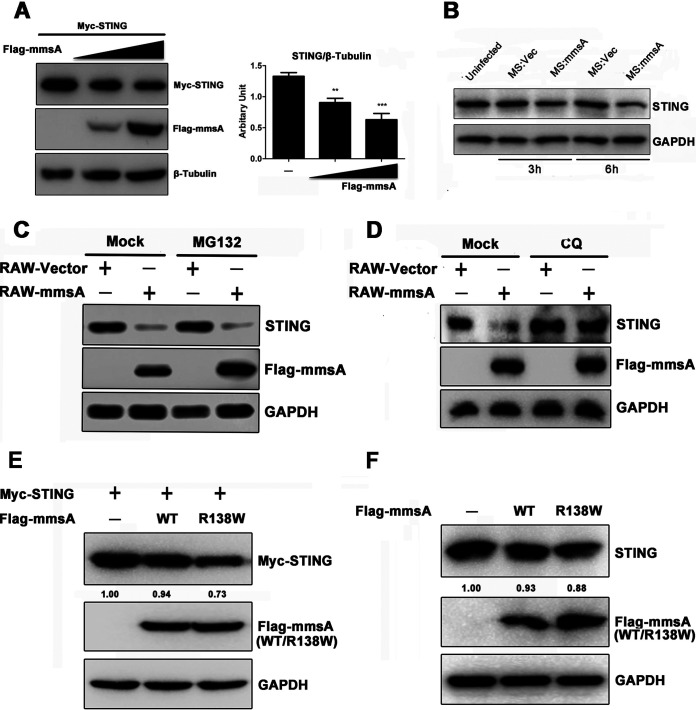
MmsA mediates autophagy-dependent degradation of STING. (A) HEK293T cells were transfected with empty vector or the plasmids expressing Flag-MmsA in increasing amounts, along with expression plasmids for Myc-STING, for 48 h. (Left) Cell lysate was subjected to SDS-PAGE followed by immunoblotting with the indicated antibodies. (Right) Quantification of the protein level of STING to β-tubulin is shown. (B) RAW264.7 cells were infected with M. smegmatis::vector and M. smegmatis::MmsA for 3 and 6 h at an MOI of 10, and cell lysates were subjected to SDS-PAGE, followed by immunoblotting with the indicated antibodies. (C) RAW-MmsA and RAW-Vector cells were treated with dimethyl sulfoxide (DMSO) or MG132 (10 μM) for 6 h and analyzed by immunoblotting with the indicated antibodies. (D) RAW-MmsA and RAW-Vector cells were treated with PBS or CQ (50 μM) for 6 h and analyzed by immunoblotting with the indicated antibodies. (E and F) HEK293T (E) or RAW264.7 (F) cells were transfected with empty vector or the plasmid expressing Flag-MmsA-WT or Flag-MmsA-R138W, along with Myc-STING expression plasmid, for 48 h. Cell lysate was subjected to SDS-PAGE followed by immunoblotting with the indicated antibodies. Numbers underneath the blot represent the fold changes of STING band intensity compared with that of the loading control, GAPDH, using Image J software.

Interestingly, we noticed that MmsA overexpression consistently reduced the level of STING protein l in HEK293T cells. This effect was confirmed to occur in a dose-dependent manner (Fig. 5A). Likewise, endogenous STING levels were decreased in M. smegmatis::MmsA-infected RAW264.7 cells compared to those in M. smegmatis::vector-infected cells (Fig. 5B). To exclude the possibility that the decrease in the STING protein level was due to reduced transcription, we performed quantitative PCR (qPCR) and found that the mRNA levels of STING had not significantly changed in MmsA-overexpressing RAW264.7 cells (Fig. S3A), suggesting that MmsA affects STING levels at the posttranslational level. To further determine the specificity of M. tuberculosis MmsA for STING degradation, we transfected the plasmid expressing MmsA homologous protein from nonpathogenic strain M. smegmatis-MmsA into HEK293T cells. Our result suggested that M. tuberculosis-MmsA, but not M. smegmatis-MmsA, enhanced STING protein degradation (Fig. S3B), indicating the specificity of M. tuberculosis-MmsA mediated STING degradation.

10.1128/mBio.03254-19.3 FIG S3 M. tuberculosis MmsA induces the degradation of STING. (A) Stable transfected cell lines RAW-MmsA and RAW-Vector were analyzed by qPCR. *STING* mRNA was measured and *GAPDH* used as a loading control. The results are shown as means ± SEM. NS, not significant. (B) HEK293T cells were transfected with empty vector or the plasmids expressing Flag-M. tuberculosis-MmsA or Flag-MS-MmsA, along with expression plasmids of pMyc-human-STING for 48 h. Cell lysate was subjected to SDS-PAGE followed by immunoblotting with the indicated antibodies. Download FIG S3, TIF file, 0.4 MB. Copyright © 2020 Sun et al. 2020 Sun et al. This content is distributed under the terms of the Creative Commons Attribution 4.0 International license.

It has been reported that STING undergoes ubiquitination and can be degraded via the proteasome-dependent pathway (45–48). Therefore, we assessed whether MmsA promoted the degradation of STING through the proteasomal pathway. However, MG132, an inhibitor of the proteasome pathway, failed to block MmsA-mediated degradation of STING in RAW-MmsA cells (Fig. 5C), indicating that MmsA did not promote STING degradation through the proteasome-dependent pathway. Next, we investigated if autophagy serves as an alternative mechanism responsible for STING degradation. As a result, we found that MmsA-mediated STING degradation was indeed inhibited by chloroquine (CQ), an autophagy inhibitor that blocks the fusion between autophagosomes and lysosomes in MmsA-expressing RAW264.7 cells (Fig. 5D). Accordingly, rapamycin, an autophagy inducer, significantly enhanced MmsA-mediated endogenous STING degradation in RAW264.7 cells (Fig. S4A). Additional expression of MmsA in STING-coexpressing 293T cells did not directly induce cell autophagy (Fig. S4B), suggesting that the observed STING reduction was not due to MmsA-induced autophagy. A previous report showed that the R138W mutation in the *mmsA* gene exists in the hypervirulent clinical Beijing-like isolates and that this mutation was considered likely to affect the protein function, as scored by PROVEAN (49). To determine whether the mutation would affect STING degradation, we constructed plasmids expressing the MmsA-R138W mutant and found that MmsA-R138W enhanced the degradation of exogenous STING in HEK293T cells (Fig. 5E) or endogenous STING in RAW264.7 cells (Fig. 5F). Taken together, these results demonstrated that MmsA reduces STING protein level through autophagy-dependent degradation.

10.1128/mBio.03254-19.4 FIG S4 M. tuberculosis MmsA mediated autophagy-dependent degradation of STING. (A) RAW-MmsA and RAW-Vector were treated with DMSO or rapamycin (100 nM) for 6 h and analyzed by immunoblotting with the indicated antibodies. (B) HEK293T cells were transfected with empty vector or the plasmids expressing Flag-MmsA, along with expression plasmids of Myc-human-STING, for 48 h. Cell lysate was subjected to SDS-PAGE followed by immunoblotting with the indicated antibodies. Download FIG S4, TIF file, 0.4 MB. Copyright © 2020 Sun et al. 2020 Sun et al. This content is distributed under the terms of the Creative Commons Attribution 4.0 International license.

### MmsA interacts with p62 to enable autophagic degradation of STING.

**FIG 6:**
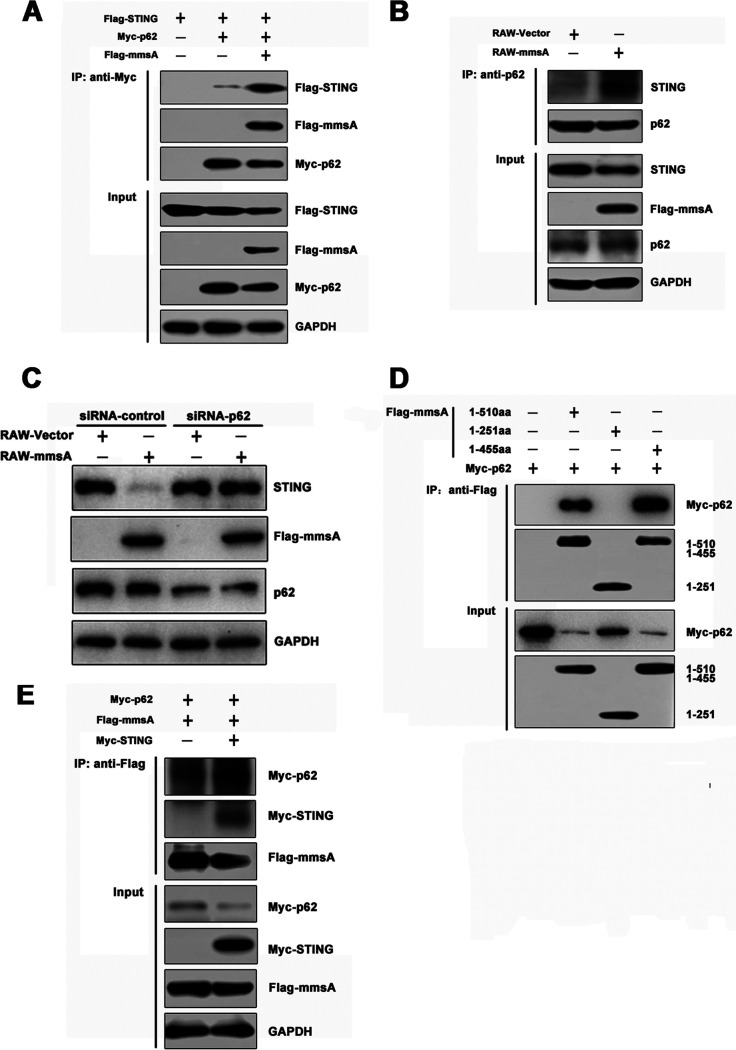
MmsA interacts with p62 for STING autophagy degradation. (A) HEK293T cells were transfected with Flag-STING and Myc-p62, with or without Flag-MmsA, for 48 h. Cell lysates were immunoprecipitated with the anti-Myc antibody, followed by immunoblotting with the indicated antibodies. (B) Cell lysates of RAW-MmsA and RAW-Vector were immunoprecipitated with the anti-p62 antibody and analyzed by immunoblotting with the indicated antibodies. (C) RAW-MmsA and RAW-Vector cells were transfected with siRNA-control and siRNA-p62 for 48 h and analyzed by immunoblotting with the indicated antibodies. (D) HEK293T cells were transfected with pFlag-MmsA or its mutants along with pMyc-p62 for 48 h. Cell lysates were immunoprecipitated with the anti-Flag antibody and then immunoblotted with the indicated antibodies. (E) HEK293T cells were transfected with pMyc-p62, pFlag-MmsA, and pMyc-human-STING or Myc-Vector for 48 h. Cell lysates were immunoprecipitated with the anti-Flag antibody and then immunoblotted with the indicated antibodies.

Emerging evidence emphasizes that p62 (encoded by *SQSTM1*) functions as a major autophagy cargo receptor that delivers proteins for selective autophagosome degradation (50, 51). Recent studies showed that p62 is essential for DNA-stimulated STING degradation (52). To investigate whether MmsA could promote the STING-p62 autophagy degradation, we performed co-IP assays in HEK293T cells and observed that MmsA expression enhanced the STING-p62 interaction (Fig. 6A). Endogenous immunoprecipitation in RAW264.7 cells also confirmed that MmsA increased the coprecipitation of STING and p62 (Fig. 6B). As MmsA could interact with STING, we hypothesized that MmsA functions as a bridge to facilitate p62-mediated autophagic degradation of STING. To further verify whether p62-mediated selective autophagy is necessary for STING degradation, we performed the p62 knockdown experiment using short interfering RNA (siRNA) in RAW-MmsA and RAW-Vector cells. Compared with the scramble siRNA control, MmsA was no longer able to promote STING degradation in the cells treated with siRNA-p62, suggesting that the degradation of STING by MmsA was indeed dependent on autophagy receptor protein p62 (Fig. 6C). To test this hypothesis, co-IPs of different truncations of MmsA and p62 were performed, and full-length MmsA and MmsA^1-455aa^ were found to interact with p62. Notably, we found that MmsA^1-251aa^ was not able to bind p62, implying that the C terminus (aa 252 to 455) of MmsA is critical for its interaction with p62 (Fig. 6D).

STING also enhanced the interaction between MmsA and p62 in HEK293T cells, suggesting that the interplay between MmsA and STING improves the binding of MmsA to p62 (Fig. 6E). Together, our results suggested that MmsA acts as a bridge that links p62 and STING to enhance p62-mediated autophagic degradation of STING.

### M. tuberculosis MmsA is inversely correlated with IFN-β production during M. tuberculosis infection.

**FIG 7:**
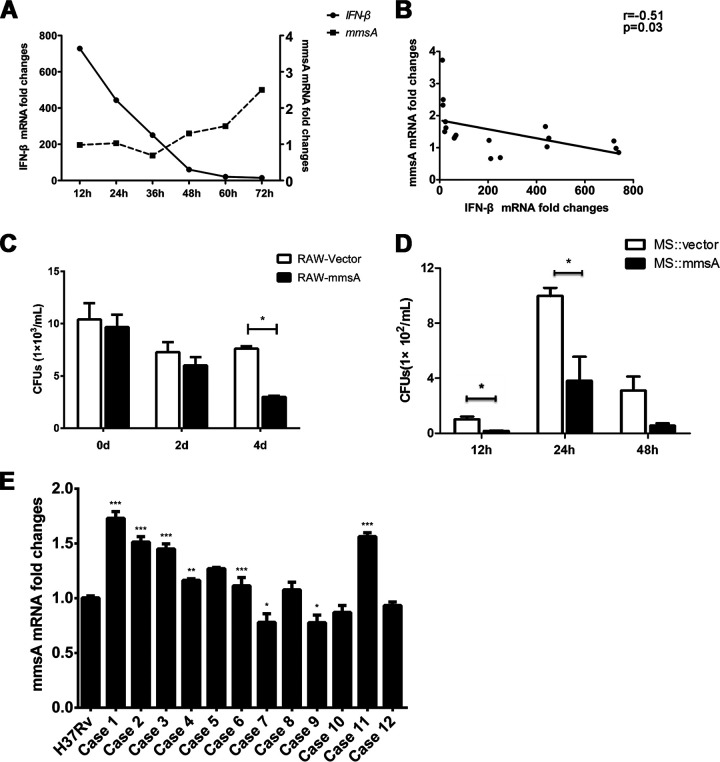
M. tuberculosis MmsA is inversely correlated with IFN-β production during M. tuberculosis infection. (A) RAW264.7 cells infected with H37Rv at an MOI of 10 for the indicated times. The mRNA levels of IFN-β and MmsA were measured by qPCR using *GAPDH* and *sigA* as internal reference controls, respectively. (B) The correlation between IFN-β and MmsA during M. tuberculosis infection was assessed with the Pearson test. (C) RAW-MmsA and RAW-Vector cells were infected with H37Rv at an MOI of 10. After 6 h, cells were washed with PBS three times, changed to medium containing 200 μg/ml amikacin, and then further cultured for 0, 2, and 4 days and lysed with 1 ml 0.1% Triton X-100. Lysates were spread on 7H11 plates to detect the bacterial number. (D) RAW264.7 cells were infected with M. smegmatis::MmsA or M. smegmatis::Vector at an MOI of 20. After 12, 24, and 48 h of infection, the macrophages were washed and lysed. Lysates were plated to detect the bacterial number. (E) The mRNA level of MmsA from the H37Rv strain and clinical isolates from tuberculosis patients (*n* = 12) was measured by qPCR using *sigA* as an internal reference control. Data are displayed as the means ± SEM from three independent experiments. *, *P < *0.05; **, *P < *0.01; ***, *P < *0.001; each by Student's *t* test.

To investigate the relationship between M. tuberculosis MmsA expression and IFN-β production during M. tuberculosis infection, we infected RAW264.7 cells with H37Rv. The levels of MmsA and IFN-β were measured by qPCR for the indicated time. The induction of IFN-β decreased gradually, whereas MmsA expression increased progressively during the infection course (Fig. 7A). An inverse correlation was also detected between IFN-β production and MmsA levels in M. tuberculosis-infected macrophages (Fig. 7B). To investigate the effect of M. tuberculosis MmsA on mycobacterial survival, we infected the RAW-Vector or RAW-MmsA cells with H37Rv and measured the number of bacterial CFU in the cell lysate. It was shown that the overexpression of MmsA in RAW264.7 cells decreased H37Rv bacterial numbers (Fig. 7C). Meanwhile, M. smegmatis with MmsA overexpression (M. smegmatis::MmsA) also reduced the intracellular bacterial numbers (Fig. 7D) in RAW264.7 cells compared with that of control M. smegmatis::Vector infection, which is in line with the previous studies that type I IFN exacerbates M. tuberculosis infection (53, 54). This may be attributed to the increased STING degradation inhibiting type I IFN responses. Additionally, we detected the MmsA mRNA levels in clinical isolates and found that they showed differential expression compared to those in the standard virulent strain M. tuberculosis H37Rv (Fig. 7E). Collectively, these data indicated that MmsA affects type I IFN production by regulating the STING level during the pathogenesis of tuberculosis.

## DISCUSSION

M. tuberculosis MmsA was previously speculated to be encoded by the class of aldehyde dehydrogenase-6, which can be regulated by the stress response sigma factor SigG under stress conditions (55, 56). This study identified that M. tuberculosis MmsA facilitates STING autophagic degradation via protein-protein interactions and modulates the STING-mediated IFN pathway, which appears to be a novel mechanism underlying M. tuberculosis-host interactions regulating the STING-type I IFN pathway.

Although STING is critical for IFN-β production during M. tuberculosis infection *in vitro* and *in vivo* (16, 17, 19–21, 57), its role in host defense against M. tuberculosis remains debatable. Several studies have reported that BMDMs from STING-deficient mice enhance better bacterial survival than BMDMs from wild-type (WT) mice (17, 19, 23). Nevertheless, another study reported that the activated STING pathway enhances the protective immunity by eliciting both the Th1 and Th17 immune responses (but not type I IFN signaling) (58). In addition, other studies have suggested that the bacterial burden does not differ between STING-deficient and WT mice, indicating that the cGAS/STING pathway is dispensable for controlling M. tuberculosis infections (17, 57). Here, we found that M. tuberculosis MmsA can target STING for type I IFN production downregulation, which might contribute to the pathogenesis of mycobacterial infection, in accordance with previous studies (59, 60).

As a central immune adaptor molecule linking DNA sensing and type I IFN responses, STING is tightly regulated to maintain immune homeostasis (61, 62). Ubiquitin-dependent STING degradation, which includes RNF5, TRIM30α, and TRIM29, has been reported (45–48). Phosphorylation (63), dephosphorylation (64), and deSUMOylation (65) are also associated with STING degradation. A mycobacterial phosphodiesterase called CdnP suppresses STING activation and the type I IFN response via the hydrolysis of bacterially derived c-di-AMP and host-derived cGAMP (24). It was shown that another regulator, ASC, a key component of the inflammasome, inhibits type I IFN production by interacting with STING and disrupting its association with TBK1 via the C terminus (60). It is interesting that STING and autophagy are closely associated during M. tuberculosis infection. Evidence showed that cGAS functions upstream of STING to activate autophagy during M. tuberculosis infection (19). Direct STING activation is necessary for the formation of autophagosomes targeting M. tuberculosis (23). In this study, we found a new mechanism of STING regulation by MmsA, a mycobacterial protein, which promotes p62-mediated selective degradation of STING. Our data demonstrated that M. tuberculosis MmsA interacted with the N terminus of STING but not the C terminus of STING. Hence, the reduction of the STING-TBK complex we observed here might be due to the degradation of STING protein rather than competition binding of MmsA to STING. Furthermore, MmsA blocks the STING-type I IFN response by facilitating p62-mediated STING degradation, which provides an additional mechanism by which autophagy-mediated degradation also reduces STING levels during M. tuberculosis infection.

It has been reported that increased type I IFNs seem to be deleterious for M. tuberculosis survival in the mouse infection model, in association with reduced Th1 immunity (6). Using comparative genomic analysis, the MmsA R138W mutation was found to be one of nine variants exclusively existing in hypervirulence Beijing-like strains such as H37Rv and Beijing classic strains (49). Our results showed that the MmsA R138W substitution induces more degradation of STING, which may weaken STING-mediated type I IFN responses. Given that MmsA is suggested to induce the maturation of DC and Th1 polarization via mitogen-activated protein kinase and NF-κB activation (30) and might represent the dormant state of mycobacteria, because it is induced remarkably in the dormancy model of M. tuberculosis infection (66, 67), we believe that the alteration of MmsA R138W in Beijing-like strains would inhibit Th1 response, favoring the persistence of the bacterium during infection. However, further studies are needed to investigate whether the reduced Th1 immunity is relevant to the inhibition of type I IFN by MmsA and explore the effect of MmsA regarding the outcome of M. tuberculosis infection *in vivo*.

**FIG 8:**
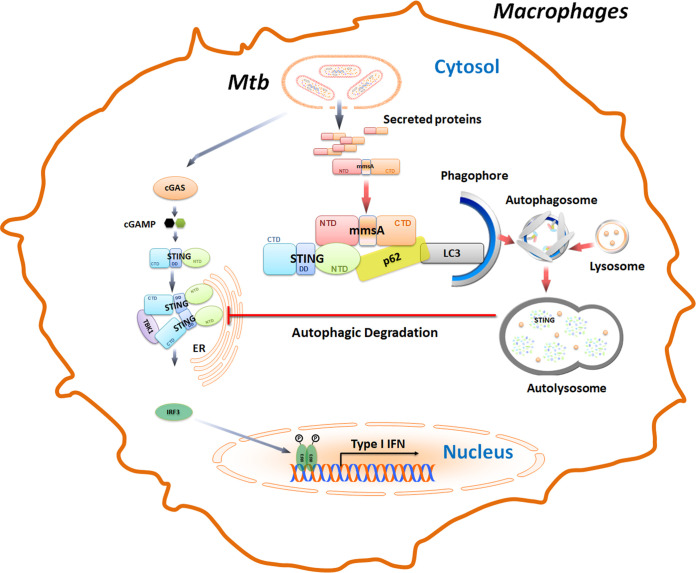
Proposed model illustrating how M. tuberculosis MmsA inhibits type I IFN responses. In M. tuberculosis-infected macrophages, M. tuberculosis releases the genomic DNA, cyclic dinucleotide, and many secreted proteins, including MmsA, into the cytosol. Mycobacterial genomic DNA, cyclic dinucleotide c-di-AMP, or host mitochondrial DNA may trigger the DNA sensor cGAS to activate the STING-TBK1-IRF3 pathway to induce IFN-β production. In this process, M. tuberculosis MmsA may act as a bridge linking p62 and STING to facilitate the p62-mediated selective autophagic degradation of STING, leading to the reduction of STING protein level and the subsequent phosphorylation of TBK1 and IRF3, which eventually blunts type I IFN responses. CTD, C-terminal domain; DD, dimerization domain; NTD, N-terminal domain.

In summary, our results show that M. tuberculosis MmsA negatively regulates type I IFN responses by facilitating the p62-mediated degradation (Fig. 8). Our results provide novel insights into a new mechanism wherein type I IFN is tightly regulated by the mycobacterial MmsA during M. tuberculosis infection. Thus, these results potentially further our understanding of the effect of different virulent strains on tuberculosis pathogenesis.

## MATERIALS AND METHODS

### Cell and bacterial culture.

HEK293T and RAW264.7 cells were maintained in Dulbecco’s modified Eagle’s medium (DMEM) supplemented with 10% fetal bovine serum (HyClone), 1% penicillin-streptomycin solution. Both cell lines were cultured in humidified air containing 5% CO_2_ at 37°C.

M. tuberculosis H37Rv and M. smegmatis mc^2^155 were grown in Middlebrook 7H9 broth medium (BD Biosciences) supplemented with 0.05% Tween 80, 0.5% glycerol, and 10% albumin-dextrose-catalase (BD Biosciences) at 37°C. The recombinant M. smegmatis expressing M. tuberculosis MmsA (M. smegmatis::MmsA) was generated by electroporating pMV261-MmsA into M. smegmatis mc^2^155. The successfully recombinant M. smegmatis was selected using kanamycin and was further confirmed by Western blotting. The control M. smegmatis::Vector was generated by electroporating pMV261 vector plasmid into M. smegmatis mc^2^155. M. smegmatis::MmsA and M. smegmatis::Vector were grown in Luria-Bertani medium supplemented with 0.05% Tween 80 and kanamycin. All mycobacteria were counted based on CFU in Middlebrook 7H11 broth medium (BD Biosciences) supplemented with 0.5% glycerol and 10% oleic acid-albumin-dextrose-catalase (BD Biosciences) at 37°C.

### Mass spectroscopy.

RAW264.7 cells were left uninfected or were infected with the M. tuberculosis virulent strain H37Rv for 6 h at a multiplicity of infection (MOI) of 10. The cells were washed with phosphate-buffered saline (PBS) and lysed with immunoprecipitation lysis buffer containing 100 μM phenylmethylsulfonyl fluoride (PMSF; Beyotime). The cell lysates were subjected to immunoprecipitation with 50 μl protein A-agarose and anti-STING (1:50) antibody (Ab) or normal rabbit IgG (1 μg/ml). The candidate proteins interacting with STING were identified by mass spectroscopy (Applied Protein Technology).

### Plasmids and siRNA transfection.

The human STING cDNA from GENECHEM, Ltd., murine STING cDNA from RAW264.7 cells, as well as the truncated human STING, aa 1 to 190, and STING, aa 191 to 379, were cloned into pMSCV-puro-Myc vector and named pMyc-human-STING, pMyc-murine-STING, pSTING^N-Region^, and pSTING^C-Region^. Human p62 cDNA from THP-1 cells was also cloned into pMSCV-puro-Myc vector and named pMyc-p62. The coding sequences of M. tuberculosis MmsA and its truncated mutants (aa 1 to 251 and 1 to 455) were amplified from H37Rv genomic DNA and cloned into pFlag-CMV2 vector to get the pFlag-MmsA, pFlag-MmsA^1-251aa^, and pFlag-MmsA^1-455aa^ plasmids. Flag-MmsA was also cloned into the retroviral expression vector pMSCV-EGFP to get pMSCV-EGFP-Flag-MmsA for the generation of retrovirus expressing Flag-MmsA. M. tuberculosis MmsA was also cloned into pMV261 and pEGFP-C1 vectors to get the plasmids pMV261-MmsA and pEGFP-MmsA. MmsA was also cloned into pMSCV-puro-Myc vector and named pMyc-MmsA. M. smegmatis MmsA was amplified using M. smegmatis genomic DNA and cloned into pFlag-CMV2 vector to generate pFlag-M. smegmatis MmsA. These plasmids were constructed using the ClonExpress II one-step cloning kit (C112-01; Vazyme) or standard molecular biology techniques. The pFlag-MmsA-R138W plasmid expressing M. tuberculosis MmsA-R138W mutant was generated by the site-directed mutagenesis method using Phanta Max super-fidelity DNA polymerase (P515-01; Vazyme). Plasmids expressing Flag-STING, Flag-TBK1, Flag-IRF3, and Flag-MAVS were gifts from Fangfang Zhou (Soochow University). The primers used for clones and subclones are provided in Table S2 in the supplemental material. For plasmid transfection, Lipofectamine 3000 (Life Technologies) and Longtrans (UCallM) were used according to the manufacturers’ manuals.

10.1128/mBio.03254-19.6 TABLE S2 List of all primers for plasmid construction. Download Table S2, DOCX file, 0.02 MB. Copyright © 2020 Sun et al. 2020 Sun et al. This content is distributed under the terms of the Creative Commons Attribution 4.0 International license.

For knockdown experiments, the siRNAs targeting to human p62 coding sequence were designed and synthesized by RiboBio. A total of 2 × 10^5^ RAW264.7 cells/well were seeded in 12-well plates and then transfected with p62 siRNA (50 nM) or control siRNA using Lipofectamine RNAiMAX (Life Technologies).

### Generation of RAW-MmsA and RAW-Vector cells.

HEK293T cells were seeded in 60-mm dishes and transfected with 2 μg of packing plasmid pCL-Ampho (a kind gift from Hui Zheng, Soochow University) and 4 μg of pMSCV-EGFP-Flag-MmsA using Lipofectamine 3000. The virus-containing medium was harvested 48 and 72 h posttransfection, centrifuged at 4°C, 3,000 rpm, for 30 min, and filtered through a 0.45-μm filter (MILLEX GP). RAW264.7 cells were infected with the above-described retrovirus in the presence of Polybrene (10 μg/ml; Sigma), and the culture plates were centrifuged at 2,500 rpm for 30 min at 30°C for high efficiency of infection. The cells were further cultured in fresh medium for 72 to ∼96 h, and the GFP^+^ cells were sorted by a FACSAria III cell sorter (BD Biosciences). High purity of GFP^+^ cells was obtained by two or three rounds of sorting and termed Raw-MmsA cells. Similarly, RAW264.7 cells transduced with MSCV-EGFP retrovirus were termed RAW-Vector as a control.

### Chemical reagents and antibodies.

Poly(deoxyadenylic-thymidylic) acid [poly(dA:dT)] (P0883) was from Sigma. MG132 (S2619), chloroquine (CQ) (C6628), and rapamycin (A606203) were from Selleck, Sigma, and Sangon Biotech, respectively.

The following antibodies were used for immunoblotting. Anti-STING (13647), anti-TBK1/NAK (3013), anti-phospho-TBK1/NAK (Ser172) (5483), anti-IRF3 (4302), anti-β-tubulin (2128), anti-His-tag (12698), and anti-Myc-tag (2276) were from Cell Signaling Technologies. Anti-Flag (F1804), anti-Myc (C3956), anti-phospho-IRF3 (pSer385) (SAB4503926), and anti-glyceraldehyde-3-phosphate dehydrogenase (GAPDH) (G9545) were from Sigma. Anti-DDDDK-tag (PM020) and anti-p62/SQSTM1 (PM045) were from MBL. Anti-calnexin (10427-2-AP) was from Proteintech. Horseradish peroxidase (HRP)-conjugated anti-rabbit IgG (4030-05) or anti-mouse (1034-05) IgG was from SouthernBiotech. Mouse polyclonal antibody against MmsA antiserum was raised against recombinant M. tuberculosis MmsA expressed by Escherichia coli.

Anti-STING (13647; Cell Signaling Technology), normal rabbit IgG (sc2027; Santa Cruz Biotechnology), protein A-agarose (11-134-515-001; Roche), cell lysis buffer for Western blotting and IP (P0013; Beyotime), EZview red anti-c-Myc affinity gel (E6654; Sigma), and EZview red anti-Flag M2 affinity gel (F2426; Sigma) were used for immunoprecipitation.

The following antibodies were used for immunofluorescence: anti-Flag (F1804; Sigma), anti-Myc (C3956; Sigma), anti-STING (ab92605; Abcam), DyLight 549-goat anti-mouse IgG (A23310; Abbkine), DyLight 488-goat anti-rabbit IgG (072-03-15-06; KPL), DyLight 488-goat anti-mouse IgG (072-03-18-06; KPL), and DyLight 549-goat anti-rabbit IgG (A23320; Abbkine).

### Western blotting and immunoprecipitation.

Cells were lysed on ice for 30 min using lysis buffer containing 100 μM PMSF and PhoStop phosphatase inhibitor (Roche). Equal amounts of protein from every sample were separated by SDS-PAGE and transferred to polyvinylidene difluoride (PVDF) membranes (Millipore). The membrane was blocked with PBS containing 0.1% Tween 20 and 5% skim milk for at least 60 min at room temperature (RT). These membranes were further incubated with the appropriate primary antibodies and HRP-conjugated secondary antibodies. The membranes were detected by Super Signal West Pico chemiluminescent substrate (Thermo Fisher Scientific), and the chemical luminesce was visualized using an Amersham Imager 600 (AI600; GE Healthcare). The intensity of the bands was quantified by ImageJ software.

For native polyacrylamide gel electrophoresis, cells were lysed in nondenatured lysis buffer (R0030; Solarbio) containing 100 μM PMSF. In brief, the 7.5% native PAGE gels were prerun with 25 mM Tris and 192 mM glycine, pH 8.4, containing 1% deoxycholate in a cathode chamber for 30 min at 70 V on ice. Samples in native loading buffer (P1017; Solarbio) were separated at 70 V for 180 min on ice and then transferred to PVDF membranes for immunoblotting.

For immunoprecipitation in HEK293T cells, 2 × 10^6^ cells were plated onto a 60-mm dish and transfected with the indicated plasmids. Forty-eight hours posttransfection, the cells were washed three times with 1 ml cold 1× PBS and lysed with 1 ml cell lysis buffer (Beyotime) containing 1% Triton X-100 and 100 μM PMSF for Western blotting and immunoprecipitation. The cell lysates then were incubated with 20 μl EZview red anti-Myc/Flag affinity gel for 4°C with overnight shaking. The Sepharose beads were eluted with 2× protein loading buffer after five washings with lysis buffer and boiled for 10 min before immunoblot analysis. For the semiendogenous co-IP, the lysates of RAW-Vector and RAW-MmsA were incubated with anti-Flag antibody and analyzed by immunoblot analysis with anti-STING antibody.

### Confocal microscopy.

To determine MmsA and STING colocalization, HEK293T cells were grown on live cell imaging culture dishes (Livefocus) and transfected with pFlag-MmsA and pMyc-murine/human-STING. The cells were washed with PBS 36 h posttransfection, fixed with ice-cold 4% paraformaldehyde, and permeabilized with 0.1% Triton X-100 PBS. The cells were then blocked with PBS containing 3% bovine serum albumin (BSA; Sigma) and incubated with anti-Flag mouse Ab and anti-Myc rabbit Ab at 4°C overnight. After washing with PBS, cells were incubated with DyLight 549-goat anti-mouse IgG and DyLight 488-goat anti-rabbit IgG (human STING) or DyLight 488-goat anti-mouse IgG and DyLight 549-goat anti-rabbit IgG (murine STING) for 1 h at RT, and the cellular DNA was stained by 4′,6-diamidino-2-phenylindole (DAPI) (Sigma) for 10 min at RT. To examine the colocalization of STING and MmsA in the context of mycobacterial infection, RAW264.7 cells were infected with M. smegmatis::MmsA or H37Rv at an MOI of 10. At 24 h postinfection, the cells were fixed and processed for the staining of anti-MmsA mouse Ab and anti-STING rabbit Ab. For subcellular localization, RAW264.7 cells were infected with M. smegmatis::MmsA for 12 h. The cells were processed as described above and immunostained with anti-calnexin rabbit antibody and anti-MmsA mouse antibody. After washing, the cells were stained with DyLight 488/549-goat anti-mouse IgG and DyLight 549/488-goat anti-rabbit IgG secondary antibodies. Colocalization images were captured using a laser scanning confocal microscope (Nikon A1).

### Luciferase reporter assay.

HEK293T cells were seeded into 12-well plates at a density of 3 × 10^5^ cells/well. The cells/well were then transfected with 500 ng the indicated plasmids, 200 ng IFN-β-Luc plasmid, and 40 ng *Renilla* luciferase vector as an internal control. The cell lysates were assayed for luciferase activities 24 h posttransfection using the Dual-Luciferase reporter assay system (E1910; Promega) according to the manufacturer’s instructions. The luminance was detected by a microplate reader (BioTek Synergy4; PerkinElmer).

### Quantitative PCR.

Total RNA was isolated using RNAiso plus (9109; TaKaRa) according to the manufacturer’s instructions and subjected to reverse transcription with a PrimeScript RT reagent kit (RR037A; TaKaRa). RNA from M. tuberculosis clinical isolates was extracted via ultrasonication for 10 min (on/off time, 5 s/5 s) before adding RNAiso plus. All gene transcripts were quantified by real-time PCR with PowerUP SYBR green master mix (A25742; Applied Biosystems) using an ABI Q6 instrument (Applied Biosystems). GAPDH was used as a reference in RAW64.7 cells, and SigA was used as an internal control for the normalization in M. tuberculosis. The primers were used for qPCR as shown in Table S3.

10.1128/mBio.03254-19.7 TABLE S3 List of primers for qPCR analysis. Download Table S3, DOCX file, 0.01 MB. Copyright © 2020 Sun et al. 2020 Sun et al. This content is distributed under the terms of the Creative Commons Attribution 4.0 International license.

### Intracellular survival detection.

For the detection of bacteria survival within macrophage, RAW-Vector and RAW-MmsA cells seeded in 6-well plates (1 × 10^5^/well) were infected with H37Rv at an MOI of 10. The infected cells were changed into DMEM containing 200 μg/ml amikacin and 1% antibiotic/antimycotic solution (Gibco) to kill extracellular bacteria. The infected cells were lysed with 1 ml sterile water containing 0.05% Triton X-100 0, 2, and 4 days postinfection. For CFU assay, 50-μl lysates were added to Middlebrook 7H10 agar plates and cultured for 3 weeks at 37°C.

RAW264.7 cells were infected with M. smegmatis::MmsA or M. smegmatis::Vector at an MOI of 20. The cells were washed and lysed 12, 24, and 48 h postinfection as described above. For CFU assay, 50-μl lysates were added to Luria-Bertani plates supplemented with kanamycin and cultured for 5 days at 37°C.

### Clinical M. tuberculosis strains from tuberculosis patients.

The RNA samples of clinical M. tuberculosis strains were obtained from the Department of Respiratory Medicine at the affiliated Hospital of Zun Yi Medical University, China. The diagnosis of active tuberculosis was based on positive Ziehl-Neelsen stain and Lowenstein-Jensen culture.

### Statistical analysis.

All data are shown as the means ± standard errors of the means (SEM) and were evaluated with a two-tailed, unpaired Student's *t* test using GraphPad Prism software 5.0. *P *values of <0.05 (*), <0.01 (**), and <0.001 (***) were considered significant. Correlations were performed using the Pearson test.

## References

[1] WHO. 2019. Global tuberculosis report 2019. World Health Organization, Geneva, Switzerland.

[2] Donovan ML, Schultz TE, Duke TJ, Blumenthal A. 2017. Type I interferons in the pathogenesis of tuberculosis: molecular drivers and immunological consequences. Front Immunol 8:1633. doi:10.3389/fimmu.2017.01633. 29230217 PMC5711827

[3] Sabir N, Hussain T, Shah SZA, Zhao D, Zhou X. 2017. IFN-beta: a contentious player in host-pathogen interaction in tuberculosis. Int J Mol Sci 18:2725. doi:10.3390/ijms18122725. PMC5751326 29258190

[4] Moreira-Teixeira L, Mayer-Barber K, Sher A, O'Garra A. 2018. Type I interferons in tuberculosis: foe and occasionally friend. J Exp Med 215:1273–1285. doi:10.1084/jem.20180325. 29666166 PMC5940272

[5] Berry MP, Graham CM, McNab FW, Xu Z, Bloch SA, Oni T, Wilkinson KA, Banchereau R, Skinner J, Wilkinson RJ, Quinn C, Blankenship D, Dhawan R, Cush JJ, Mejias A, Ramilo O, Kon OM, Pascual V, Banchereau J, Chaussabel D, O'Garra A. 2010. An interferon-inducible neutrophil-driven blood transcriptional signature in human tuberculosis. Nature 466:973–977. doi:10.1038/nature09247. 20725040 PMC3492754

[6] Manca C, Tsenova L, Freeman S, Barczak AK, Tovey M, Murray PJ, Barry C, Kaplan G. 2005. Hypervirulent M. tuberculosis W/Beijing strains upregulate type I IFNs and increase expression of negative regulators of the Jak-Stat pathway. J Interferon Cytokine Res 25:694–701. doi:10.1089/jir.2005.25.694. 16318583

[7] Manca C, Tsenova L, Bergtold A, Freeman S, Tovey M, Musser JM, Barry CER, Freedman VH, Kaplan G. 2001. Virulence of a Mycobacterium tuberculosis clinical isolate in mice is determined by failure to induce Th1 type immunity and is associated with induction of IFN-alpha/beta. Proc Natl Acad Sci U S A 98:5752–5757. doi:10.1073/pnas.091096998. 11320211 PMC33285

[8] Matsuoka S, Fujikawa H, Hasegawa H, Ochiai T, Watanabe Y, Moriyama M. 2016. Onset of tuberculosis from a pulmonary latent tuberculosis infection during antiviral triple therapy for chronic hepatitis C. Intern Med 55:2011–2017. doi:10.2169/internalmedicine.55.6448. 27477407

[9] de Oliveira Uehara SN, Emori CT, Perez RM, Mendes-Correa MC, de Souza Paiva Ferreira A, de Castro Amaral Feldner AC, Silva AE, Filho RJ, de Souza ESIS, Ferraz ML. 2016. High incidence of tuberculosis in patients treated for hepatitis C chronic infection. Braz J Infect Dis 20:205–209. doi:10.1016/j.bjid.2015.12.003. 26867472 PMC9427596

[10] Desvignes L, Wolf AJ, Ernst JD. 2012. Dynamic roles of type I and type II IFNs in early infection with Mycobacterium tuberculosis. J Immunol 188:6205–6215. doi:10.4049/jimmunol.1200255. 22566567 PMC3370955

[11] Ward CM, Jyonouchi H, Kotenko SV, Smirnov SV, Patel R, Aguila H, McSherry G, Dashefsky B, Holland SM. 2007. Adjunctive treatment of disseminated Mycobacterium avium complex infection with interferon alpha-2b in a patient with complete interferon-gamma receptor R1 deficiency. Eur J Pediatr 166:981–985. doi:10.1007/s00431-006-0339-1. 17120031

[12] Bax HI, Freeman AF, Ding L, Hsu AP, Marciano B, Kristosturyan E, Jancel T, Spalding C, Pechacek J, Olivier KN, Barnhart LA, Boris L, Frein C, Claypool RJ, Anderson V, Zerbe CS, Holland SM, Sampaio EP. 2013. Interferon alpha treatment of patients with impaired interferon gamma signaling. J Clin Immunol 33:991–1001. doi:10.1007/s10875-013-9882-5. 23512243 PMC4136390

[13] Ishikawa H, Barber GN. 2008. STING is an endoplasmic reticulum adaptor that facilitates innate immune signalling. Nature 455:674–678. doi:10.1038/nature07317. 18724357 PMC2804933

[14] Barber GN. 2015. STING: infection, inflammation and cancer. Nat Rev Immunol 15:760–770. doi:10.1038/nri3921. 26603901 PMC5004891

[15] Hopfner KP, Hornung V. 2020. Molecular mechanisms and cellular functions of cGAS-STING signalling. Nat Rev Mol Cell Biol 21:501–521. doi:10.1038/s41580-020-0244-x. 32424334

[16] Manzanillo PS, Shiloh MU, Portnoy DA, Cox JS. 2012. Mycobacterium tuberculosis activates the DNA-dependent cytosolic surveillance pathway within macrophages. Cell Host Microbe 11:469–480. doi:10.1016/j.chom.2012.03.007. 22607800 PMC3662372

[17] Collins AC, Cai H, Li T, Franco LH, Li XD, Nair VR, Scharn CR, Stamm CE, Levine B, Chen ZJ, Shiloh MU. 2015. Cyclic GMP-AMP synthase is an innate immune DNA sensor for Mycobacterium tuberculosis. Cell Host Microbe 17:820–828. doi:10.1016/j.chom.2015.05.005. 26048137 PMC4499468

[18] Wassermann R, Gulen MF, Sala C, Perin SG, Lou Y, Rybniker J, Schmid-Burgk JL, Schmidt T, Hornung V, Cole ST, Ablasser A. 2015. Mycobacterium tuberculosis differentially activates cGAS- and inflammasome-dependent intracellular immune responses through ESX-1. Cell Host Microbe 17:799–810. doi:10.1016/j.chom.2015.05.003. 26048138

[19] Watson RO, Bell SL, MacDuff DA, Kimmey JM, Diner EJ, Olivas J, Vance RE, Stallings CL, Virgin HW, Cox JS. 2015. The cytosolic sensor cGAS detects Mycobacterium tuberculosis DNA to induce type I interferons and activate autophagy. Cell Host Microbe 17:811–819. doi:10.1016/j.chom.2015.05.004. 26048136 PMC4466081

[20] Wiens KE, Ernst JD. 2016. The mechanism for type I interferon induction by Mycobacterium tuberculosis is bacterial strain-dependent. PLoS Pathog 12:e1005809. doi:10.1371/journal.ppat.1005809. 27500737 PMC4976988

[21] Dey B, Dey RJ, Cheung LS, Pokkali S, Guo H, Lee JH, Bishai WR. 2015. A bacterial cyclic dinucleotide activates the cytosolic surveillance pathway and mediates innate resistance to tuberculosis. Nat Med 21:401–406. doi:10.1038/nm.3813. 25730264 PMC4390473

[22] Dey RJ, Dey B, Singh AK, Praharaj M, Bishai W. 2019. BCG overexpressing an endogenous STING agonist provides enhanced protection against pulmonary tuberculosis. J Infect Dis 221:1048–1056. doi:10.1093/infdis/jiz116. PMC7931846 30901058

[23] Watson RO, Manzanillo PS, Cox JS. 2012. Extracellular M. tuberculosis DNA targets bacteria for autophagy by activating the host DNA-sensing pathway. Cell 150:803–815. doi:10.1016/j.cell.2012.06.040. 22901810 PMC3708656

[24] Dey RJ, Dey B, Zheng Y, Cheung LS, Zhou J, Sayre D, Kumar P, Guo H, Lamichhane G, Sintim HO, Bishai WR. 2017. Inhibition of innate immune cytosolic surveillance by an M. tuberculosis phosphodiesterase. Nat Chem Biol 13:210–217. doi:10.1038/nchembio.2254. 28106876

[25] Cole ST, Brosch R, Parkhill J, Garnier T, Churcher C, Harris D, Gordon SV, Eiglmeier K, Gas S, Barry CER, Tekaia F, Badcock K, Basham D, Brown D, Chillingworth T, Connor R, Davies R, Devlin K, Feltwell T, Gentles S, Hamlin N, Holroyd S, Hornsby T, Jagels K, Krogh A, McLean J, Moule S, Murphy L, Oliver K, Osborne J, Quail MA, Rajandream MA, Rogers J, Rutter S, Seeger K, Skelton J, Squares R, Squares S, Sulston JE, Taylor K, Whitehead S, Barrell BG. 1998. Deciphering the biology of Mycobacterium tuberculosis from the complete genome sequence. Nature 393:537–544. doi:10.1038/31159. 9634230

[26] Liu F, Yang M, Wang X, Yang S, Gu J, Zhou J, Zhang XE, Deng J, Ge F. 2014. Acetylome analysis reveals diverse functions of lysine acetylation in Mycobacterium tuberculosis. Mol Cell Proteomics 13:3352–3366. doi:10.1074/mcp.M114.041962. 25180227 PMC4256489

[27] Kai-Cheen A, Lay-Harn G. 2018. Comparison of aqueous soluble proteins profile of Mycobacterium tuberculosis H37Rv and H37Ra and a Malaysian clinical isolate. Biotechnol Appl Biochem 65:876–882. doi:10.1002/bab.1687. 30132993

[28] Zhang YX, Tang L, Hutchinson CR. 1996. Cloning and characterization of a gene (msdA) encoding methylmalonic acid semialdehyde dehydrogenase from Streptomyces coelicolor. J Bacteriol 178:490–495. doi:10.1128/jb.178.2.490-495.1996. 8550471 PMC177683

[29] Goodwin GW, Rougraff PM, Davis EJ, Harris RA. 1989. Purification and characterization of methylmalonate-semialdehyde dehydrogenase from rat liver. Identity to malonate-semialdehyde dehydrogenase. J Biol Chem 264:14965–14971. 2768248

[30] Kim JS, Kim WS, Choi HH, Kim HM, Kwon KW, Han SJ, Cha SB, Cho SN, Koh WJ, Shin SJ. 2015. Mycobacterium tuberculosis MmsA, a novel immunostimulatory antigen, induces dendritic cell activation and promotes Th1 cell-type immune responses. Cell Immunol 298:115–125. doi:10.1016/j.cellimm.2015.10.005. 26507911

[31] Pathakumari B, Prabhavathi M, Raja A. 2015. Evaluation of cytokine and chemokine response elicited by Rv2204c and Rv0753c to detect latent tuberculosis infection. Cytokine 76:496–504. doi:10.1016/j.cyto.2015.07.028. 26298037

[32] Pathakumari B, Prabhavathi M, Anbarasu D, Paramanandhan P, Raja A. 2016. Dynamic IgG antibody response to immunodominant antigens of M. tuberculosis for active TB diagnosis in high endemic settings. Clin Chim Acta 461:25–33. doi:10.1016/j.cca.2016.06.033. 27370403

[33] Pathakumari B, Devasundaram S, Maddineni P, Raja A. 2018. Rv2204c, Rv0753c and Rv0009 antigens specific T cell responses in latent and active TB–a flow cytometry-based analysis. Int J Med Microbiol 308:297–305. doi:10.1016/j.ijmm.2017.12.001. 29325881

[34] Pathakumari B, Devasundaram S, Raja A. 2018. Altered expression of antigen-specific memory and regulatory T-cell subsets differentiate latent and active tuberculosis. Immunology 153:325–336. doi:10.1111/imm.12833. 28881482 PMC5795181

[35] Morchikh M, Cribier A, Raffel R, Amraoui S, Cau J, Severac D, Dubois E, Schwartz O, Bennasser Y, Benkirane M. 2017. HEXIM1 and NEAT1 long non-coding RNA form a multi-subunit complex that regulates DNA-mediated innate immune response. Mol Cell 67:387–399. doi:10.1016/j.molcel.2017.06.020. 28712728

[36] Sun W, Li Y, Chen L, Chen H, You F, Zhou X, Zhou Y, Zhai Z, Chen D, Jiang Z. 2009. ERIS, an endoplasmic reticulum IFN stimulator, activates innate immune signaling through dimerization. Proc Natl Acad Sci U S A 106:8653–8658. doi:10.1073/pnas.0900850106. 19433799 PMC2689030

[37] Nitta S, Sakamoto N, Nakagawa M, Kakinuma S, Mishima K, Kusano-Kitazume A, Kiyohashi K, Murakawa M, Nishimura-Sakurai Y, Azuma S, Tasaka-Fujita M, Asahina Y, Yoneyama M, Fujita T, Watanabe M. 2013. Hepatitis C virus NS4B protein targets STING and abrogates RIG-I-mediated type I interferon-dependent innate immunity. Hepatology 57:46–58. doi:10.1002/hep.26017. 22911572

[38] Bashiri G, Baker EN. 2015. Production of recombinant proteins in Mycobacterium smegmatis for structural and functional studies. Protein Sci 24:1–10. doi:10.1002/pro.2584. PMC4489920 25303009

[39] Gao P, Ascano M, Zillinger T, Wang W, Dai P, Serganov AA, Gaffney BL, Shuman S, Jones RA, Deng L, Hartmann G, Barchet W, Tuschl T, Patel DJ. 2013. Structure-function analysis of STING activation by c[G(2',5')pA(3',5')p] and targeting by antiviral DMXAA. Cell 154:748–762. doi:10.1016/j.cell.2013.07.023. 23910378 PMC4386733

[40] Pollock AJ, Zaver SA, Woodward JJ. 2020. A STING-based biosensor affords broad cyclic dinucleotide detection within single living eukaryotic cells. Nat Commun 11:3533. doi:10.1038/s41467-020-17228-y. 32669552 PMC7363834

[41] Huang YH, Liu XY, Du XX, Jiang ZF, Su XD. 2012. The structural basis for the sensing and binding of cyclic di-GMP by STING. Nat Struct Mol Biol 19:728–730. doi:10.1038/nsmb.2333. 22728659

[42] Zhou Q, Lin H, Wang S, Wang S, Ran Y, Liu Y, Ye W, Xiong X, Zhong B, Shu HB, Wang YY. 2014. The ER-associated protein ZDHHC1 is a positive regulator of DNA virus-triggered, MITA/STING-dependent innate immune signaling. Cell Host Microbe 16:450–461. doi:10.1016/j.chom.2014.09.006. 25299331

[43] Luo WW, Li S, Li C, Lian H, Yang Q, Zhong B, Shu HB. 2016. iRhom2 is essential for innate immunity to DNA viruses by mediating trafficking and stability of the adaptor STING. Nat Immunol 17:1057–1066. doi:10.1038/ni.3510. 27428826

[44] Tanaka Y, Chen ZJ. 2012. STING specifies IRF3 phosphorylation by TBK1 in the cytosolic DNA signaling pathway. Sci Signal 5:ra20. doi:10.1126/scisignal.2002521. 22394562 PMC3549669

[45] Zhong B, Zhang L, Lei C, Li Y, Mao AP, Yang Y, Wang YY, Zhang XL, Shu HB. 2009. The ubiquitin ligase RNF5 regulates antiviral responses by mediating degradation of the adaptor protein MITA. Immunity 30:397–407. doi:10.1016/j.immuni.2009.01.008. 19285439

[46] Wang Y, Lian Q, Yang B, Yan S, Zhou H, He L, Lin G, Lian Z, Jiang Z, Sun B. 2015. TRIM30alpha is a negative-feedback regulator of the intracellular DNA and DNA virus-triggered response by targeting STING. PLoS Pathog 11:e1005012. doi:10.1371/journal.ppat.1005012. 26114947 PMC4482643

[47] Xing J, Zhang A, Zhang H, Wang J, Li XC, Zeng MS, Zhang Z. 2017. TRIM29 promotes DNA virus infections by inhibiting innate immune response. Nat Commun 8:945. doi:10.1038/s41467-017-00101-w. 29038422 PMC5643338

[48] Li Q, Lin L, Tong Y, Liu Y, Mou J, Wang X, Wang X, Gong Y, Zhao Y, Liu Y, Zhong B, Dai L, Wei YQ, Zhang H, Hu H. 2018. TRIM29 negatively controls antiviral immune response through targeting STING for degradation. Cell Discov 4:13. doi:10.1038/s41421-018-0010-9. 29581886 PMC5859251

[49] Rodriguez-Castillo JG, Pino C, Nino LF, Rozo JC, Llerena-Polo C, Parra-Lopez CA, Tauch A, Murcia-Aranguren MI. 2017. Comparative genomic analysis of Mycobacterium tuberculosis Beijing-like strains revealed specific genetic variations associated with virulence and drug resistance. Infect Genet Evol 54:314–323. doi:10.1016/j.meegid.2017.07.022. 28734764

[50] Wang DW, Peng ZJ, Ren GF, Wang GX. 2015. The different roles of selective autophagic protein degradation in mammalian cells. Oncotarget 6:37098–37116. doi:10.18632/oncotarget.5776. 26415220 PMC4741918

[51] Dikic I. 2017. Proteasomal and autophagic degradation systems. Annu Rev Biochem 86:193–224. doi:10.1146/annurev-biochem-061516-044908. 28460188

[52] Prabakaran T, Bodda C, Krapp C, Zhang B‐C, Christensen MH, Sun C, Reinert L, Cai Y, Jensen SB, Skouboe MK, Nyengaard JR, Thompson CB, Lebbink RJ, Sen GC, Loo G, Nielsen R, Komatsu M, Nejsum LN, Jakobsen MR, Gyrd‐Hansen M, Paludan SR. 2018. Attenuation of cGAS-STING signaling is mediated by a p62/SQSTM1-dependent autophagy pathway activated by TBK1. EMBO J 37:e97858. 29496741 10.15252/embj.201797858 PMC5897779

[53] Dorhoi A, Yeremeev V, Nouailles G, Weiner J, Jörg S, Heinemann E, Oberbeck-Müller D, Knaul JK, Vogelzang A, Reece ST, Hahnke K, Mollenkopf H-J, Brinkmann V, Kaufmann SHE. 2014. Type I IFN signaling triggers immunopathology in tuberculosis-susceptible mice by modulating lung phagocyte dynamics. Eur J Immunol 44:2380–2393. doi:10.1002/eji.201344219. 24782112 PMC4298793

[54] Mayer-Barber KD, Andrade BB, Barber DL, Hieny S, Feng CG, Caspar P, Oland S, Gordon S, Sher A. 2011. Innate and adaptive interferons suppress IL-1alpha and IL-1beta production by distinct pulmonary myeloid subsets during Mycobacterium tuberculosis infection. Immunity 35:1023–1034. doi:10.1016/j.immuni.2011.12.002. 22195750 PMC3246221

[55] Kim CY, Webster C, Roberts JK, Moon JH, Alipio Lyon EZ, Kim H, Yu M, Hung LW, Terwilliger TC. 2009. Analysis of nucleoside-binding proteins by ligand-specific elution from dye resin: application to Mycobacterium tuberculosis aldehyde dehydrogenases. J Struct Funct Genomics 10:291–301. doi:10.1007/s10969-009-9073-z. 19911309 PMC2780684

[56] Lee JH, Geiman DE, Bishai WR. 2008. Role of stress response sigma factor SigG in Mycobacterium tuberculosis. J Bacteriol 190:1128–1133. doi:10.1128/JB.00511-07. 18039768 PMC2223564

[57] Marinho FV, Benmerzoug S, Rose S, Campos PC, Marques JT, Bafica A, Barber G, Ryffel B, Oliveira SC, Quesniaux VFJ. 2018. The cGAS/STING pathway is important for dendritic cell activation but is not essential to induce protective immunity against Mycobacterium tuberculosis infection. J Innate Immun 10:239–252. doi:10.1159/000488952. 29791904 PMC6757162

[58] Van Dis E, Sogi KM, Rae CS, Sivick KE, Surh NH, Leong ML, Kanne DB, Metchette K, Leong JJ, Bruml JR, Chen V, Heydari K, Cadieux N, Evans T, McWhirter SM, Dubensky TW, Jr, Portnoy DA, Stanley SA. 2018. STING-activating adjuvants elicit a Th17 immune response and protect against Mycobacterium tuberculosis infection. Cell Rep 23:1435–1447. doi:10.1016/j.celrep.2018.04.003. 29719256 PMC6003617

[59] Teles RM, Graeber TG, Krutzik SR, Montoya D, Schenk M, Lee DJ, Komisopoulou E, Kelly-Scumpia K, Chun R, Iyer SS, Sarno EN, Rea TH, Hewison M, Adams JS, Popper SJ, Relman DA, Stenger S, Bloom BR, Cheng G, Modlin RL. 2013. Type I interferon suppresses type II interferon-triggered human anti-mycobacterial responses. Science 339:1448–1453. doi:10.1126/science.1233665. 23449998 PMC3653587

[60] Yan S, Shen H, Lian Q, Jin W, Zhang R, Lin X, Gu W, Sun X, Meng G, Tian Z, Chen ZW, Sun B. 2018. Deficiency of the AIM2-ASC signal uncovers the STING-driven overreactive response of type I IFN and reciprocal depression of protective IFN-gamma immunity in mycobacterial infection. J Immunol 200:1016–1026. doi:10.4049/jimmunol.1701177. 29255077 PMC6309432

[61] Chen Q, Sun L, Chen ZJ. 2016. Regulation and function of the cGAS-STING pathway of cytosolic DNA sensing. Nat Immunol 17:1142–1149. doi:10.1038/ni.3558. 27648547

[62] Li Y, Wilson HL, Kiss-Toth E. 2017. Regulating STING in health and disease. J Inflamm 14:11. doi:10.1186/s12950-017-0159-2. PMC5463399 28596706

[63] Konno H, Konno K, Barber GN. 2013. Cyclic dinucleotides trigger ULK1 (ATG1) phosphorylation of STING to prevent sustained innate immune signaling. Cell 155:688–698. doi:10.1016/j.cell.2013.09.049. 24119841 PMC3881181

[64] Xia T, Yi XM, Wu X, Shang J, Shu HB. 2019. PTPN1/2-mediated dephosphorylation of MITA/STING promotes its 20S proteasomal degradation and attenuates innate antiviral response. Proc Natl Acad Sci U S A 116:20063–20069. doi:10.1073/pnas.1906431116. 31527250 PMC6778251

[65] Hu MM, Yang Q, Xie XQ, Liao CY, Lin H, Liu TT, Yin L, Shu HB. 2016. Sumoylation promotes the stability of the DNA sensor cGAS and the adaptor STING to regulate the kinetics of response to DNA virus. Immunity 45:555–569. doi:10.1016/j.immuni.2016.08.014. 27637147

[66] Hampshire T, Soneji S, Bacon J, James BW, Hinds J, Laing K, Stabler RA, Marsh PD, Butcher PD. 2004. Stationary phase gene expression of Mycobacterium tuberculosis following a progressive nutrient depletion: a model for persistent organisms? Tuberculosis 84:228–238. doi:10.1016/j.tube.2003.12.010. 15207492 PMC3195342

[67] Voskuil MI. 2004. Mycobacterium tuberculosis gene expression during environmental conditions associated with latency. Tuberculosis 84:138–143. doi:10.1016/j.tube.2003.12.008. 15207483

