# Supplementary figures and images for: Mycobacterium tuberculosis MmsA (Rv0753c) Interacts with STING and Blunts the Type I Interferon Response
Source: mBio. 2020 Dec 1;11(6):e03254-19. doi: 10.1128/mBio.03254-19 (PMC7733952; doi:10.1128/mBio.03254-19)

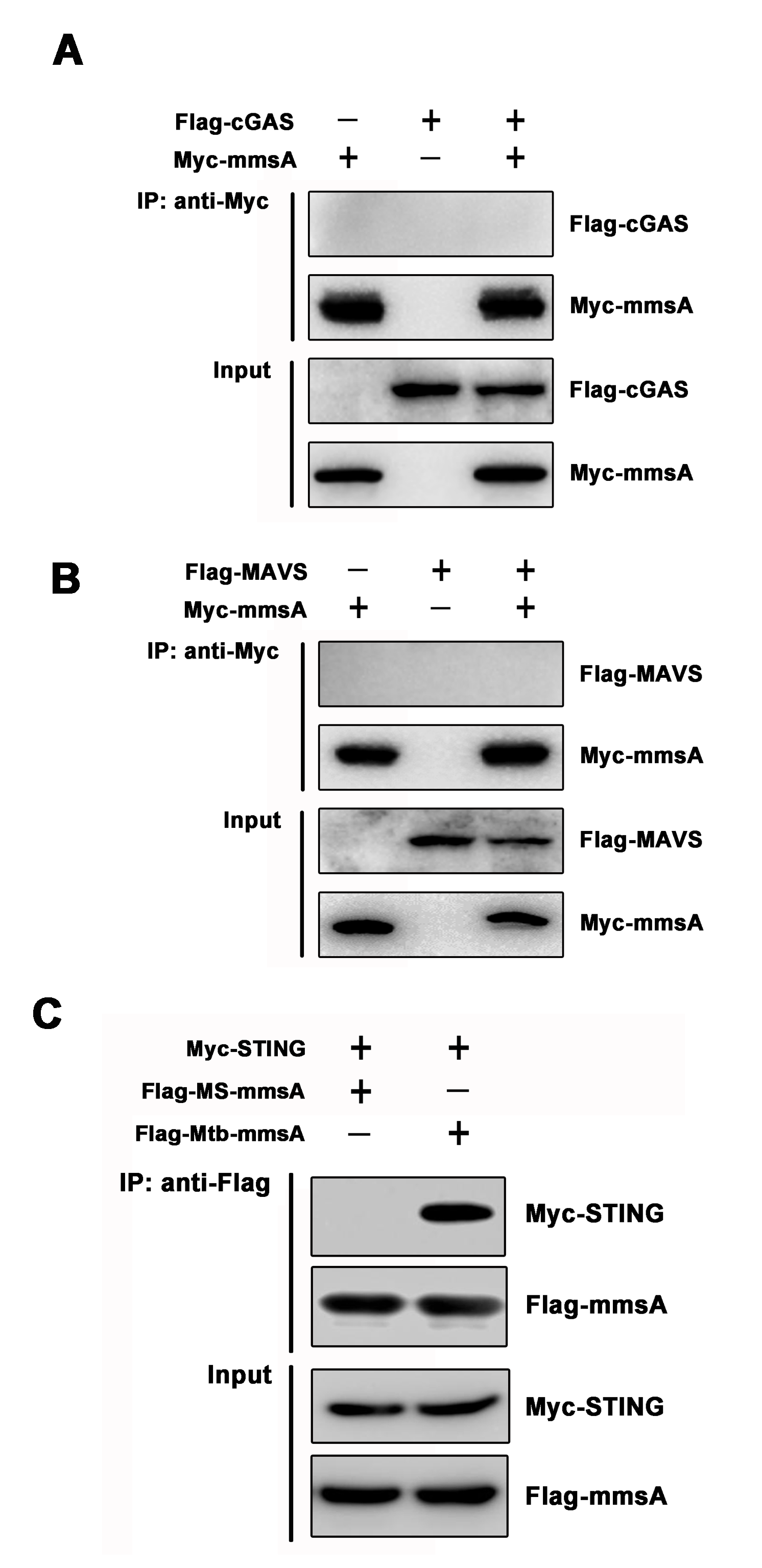

Supplement: FIG S1 [file mBio.03254-19-sf001.tif]

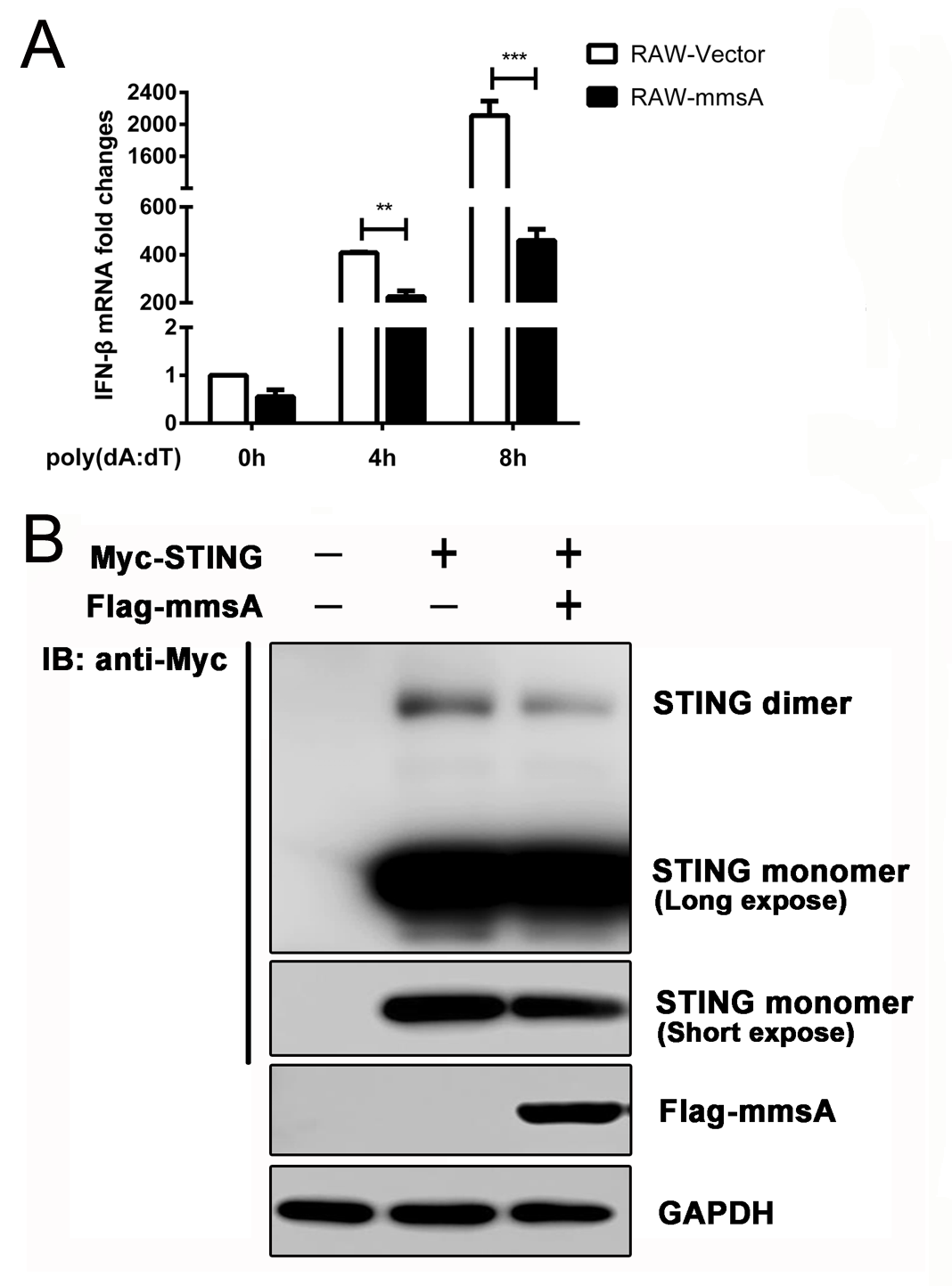

Supplement: FIG S2 [file mBio.03254-19-sf002.tif]

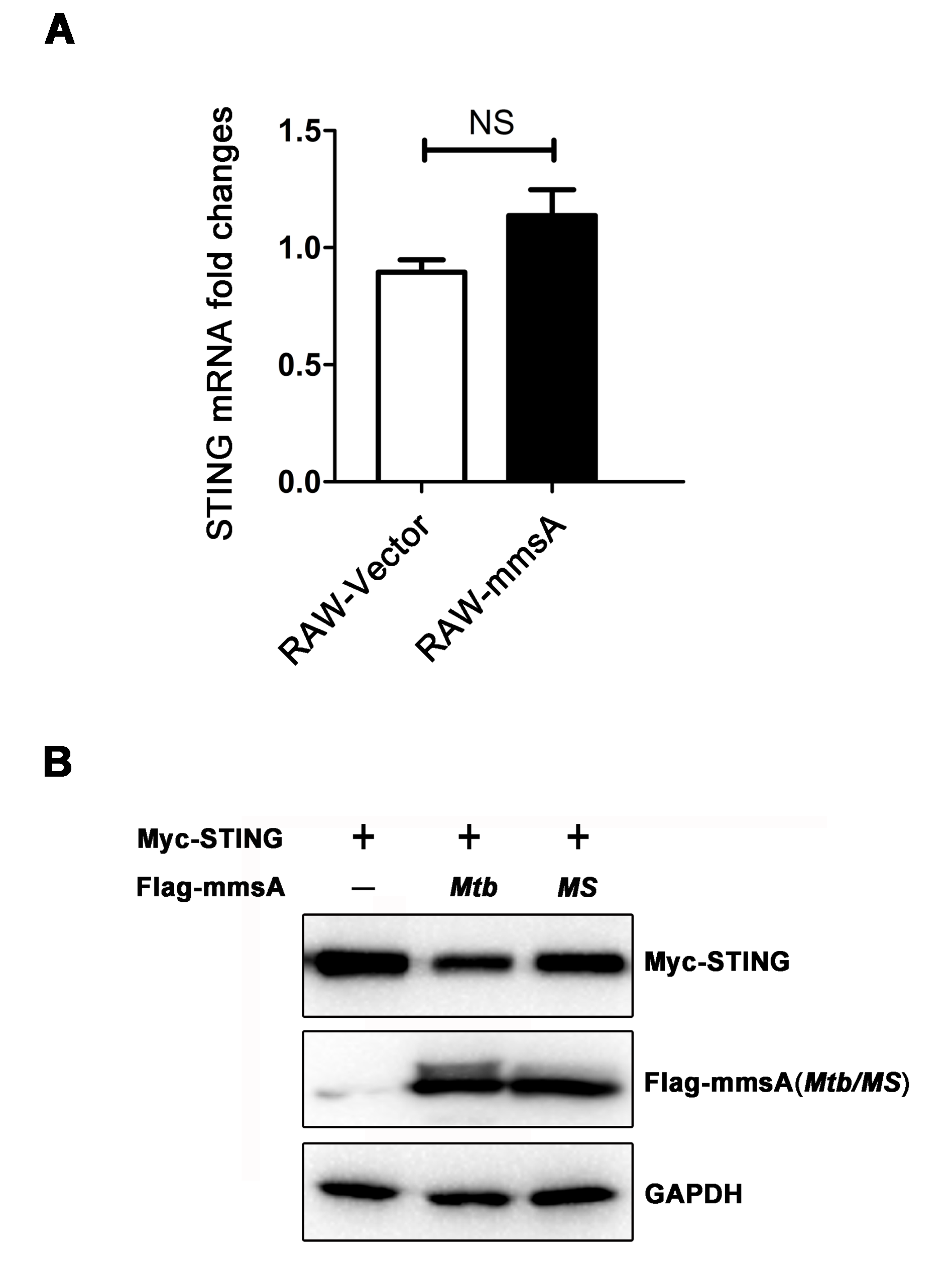

Supplement: FIG S3 [file mBio.03254-19-sf003.tif]

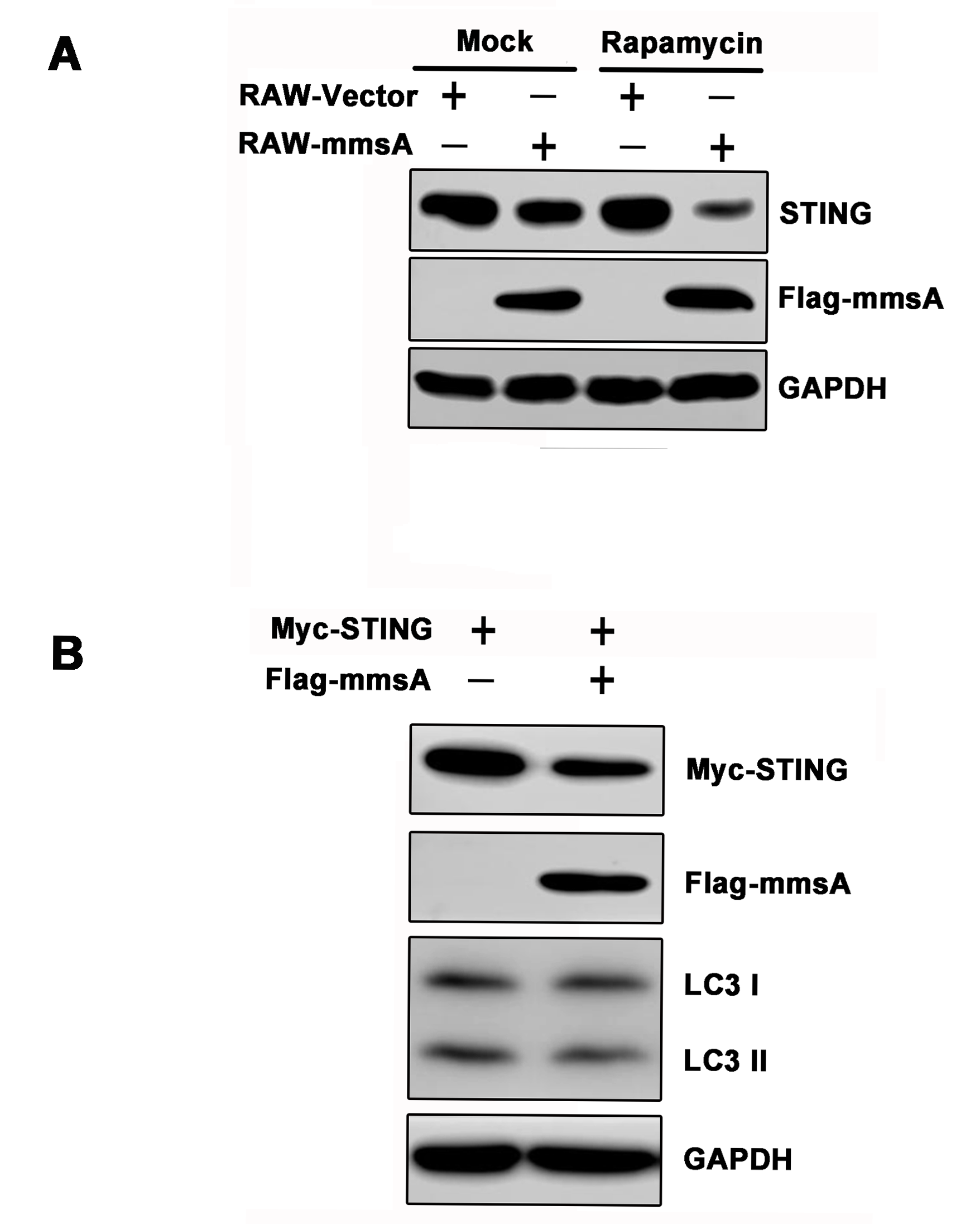

Supplement: FIG S4 [file mBio.03254-19-sf004.tif]
