# Supplementary material for: Mycobacterium tuberculosis MmsA (Rv0753c) Interacts with STING and Blunts the Type I Interferon Response
Source: mBio. 2020 Dec 1;11(6):e03254-19. doi: 10.1128/mBio.03254-19 (PMC7733952; doi:10.1128/mBio.03254-19)
Supplement: TABLE S1 [file mBio.03254-19-st001.docx]

**Table S1. Candidate STING interacting proteins identified by IP-MS approach.**

| Identified Proteins | | | | Coverage |
| --- | --- | --- | --- | --- |
| Accession | Description | Gene Name | Species |  |
| O53816 | Probable methylmalonate-semialdehyde dehydrogenase MmsA | mmsA | *Mycobacterium tuberculosis* | 49.98% |
| P35710 | Transcription factor SOX-5 | Sox5 | *Mus musculus* | 16.75% |
| Q8VIJ6 | Splicing factor, proline- and glutamine-rich | Sfpq | *Mus musculus* | 15.79% |
| Q8BPC3 | TPR_REGION domain-containing protein | Ifit3b | *Mus musculus* | 8.01% |
| O35490 | Betaine-homocysteine S-methyltransferase 1 | Bhmt | *Mus musculus* | 3.93% |
| O08792 | Transcription factor COE2 | Ebf2 | *Mus musculus* | 2.26% |
