# Supplementary material for: Mycobacterium tuberculosis MmsA (Rv0753c) Interacts with STING and Blunts the Type I Interferon Response
Source: mBio. 2020 Dec 1;11(6):e03254-19. doi: 10.1128/mBio.03254-19 (PMC7733952; doi:10.1128/mBio.03254-19)
Supplement: TABLE S2 [file mBio.03254-19-st002.docx]

**Table S2: List of all primers for Plasmids Construction**

| **Primer** | **Sequence** |
| --- | --- |
| Myc-huSTING-F | CCGGAATTCCCCCCACTCCAGCCTGCAT |
| Myc-huSTING-R | CGCGGATCCTCAAGAGAAATCCGTGCGGA |
| Myc-muSTING-F | CCGGAATTCCccatactccaacctgcatcca |
| Myc-muSTING-R | GCGTCGACtcagatgaggtcagtgcgga |
| Flag-mmsA-F (pFlag-CMV2) | AAGCTTACCACACAGATTTCACATTTCAT |
| Flag-mmsA-R (pFlag-CMV2) | TCTAGACTAACTCATTGTGGGGATGACGAA |
| Flag-mmsA-F(pMSCV-EGFP) | CTCGAGATGGATTACAAGGATGACGACGATAAG |
|  | ATGACCACACAGATTTCACATTTCAT |
| Flag-mmsA-R(pMSCV-EGFP) | GTTAACCTAACTCATTGTGGGGATGACGAA |
| STING-1-190aa-F | CCGGAATTCCccccactccagcctgcat |
| STING-1-190aa-R | CGCGGATCCtcatagcaggttgttgtaatgctg |
| STING-191-379aa-F | CCGGAATTCCcggggtgcagtgagccag |
| STING-191-379aa-R | CGCGGATCCtcaagagaaatccgtgcgga |
| mmsA-1-251aa-F | aaagacgatgacgacaagcttACCACACAGATTTCACATTTCATCG |
| mmsA-1-251aa-R | tgccacccgggatcc tctaga CTA GGCACCGCCGAAACACTG |
| mmsA-1-455aa-F | AAGCTTACCACACAGATTTCACATTTCATCGACGG |
| mmsA-1-455aa-R | TCTAGActacaccgggatcggcacgttga |
| Flag-mmsA-F (pMV-261) | GgccaagacaattgcggatccATGGATTACAAGG |
|  | ATGACGACGATAAGACCACACAGATTTCACATTTCA |
| Flag-mmsA-R (pMV-261) | tacgtcgacatcgataagcttCTAACTCATTGTGGGGATGACGA |
| Myc-mmsA-F | GCGGCCGCGACCACACAGATTTCACATTTCA |
| Myc-mmsA-R | CGCGGATCCCTAACTCATTGTGGGGATGAC |
| EGFP-mmsA | GGAAGATCTATGACCACACAGATTTCACATTTCAT |
| EGFP-mmsA | AAGCTTGCTAACTCATTGTGGGGATGACGAA |
| Myc-p62-F | CCGGAATTCCgcgtcgctcaccgtgaaggccta |
| Myc-p62-R | CGCGTCGACtcacaacggcgggggatgctttga |
| Flag-mmsA-R138W-F | ATCGACGTCTATTCGCTGTGGCAGCCCCTGGGTGTGGTCGCGGGG |
| Flag-mmsA-R138W-R | CCCCGCGACCACACCCAGGGGCTGCCACAGCGAATAGACGTCGAT |
| Flag-mmsA-MS-mmsA-F | aaagacgatgacgacaagcttACCACACAGATCCAGCACTTCA |
| Flag-mmsA-MS-mmsA-R | tgccacccgggatcctctagaCTACTTCATGGTCGGGATGACG |
