# Supplementary material for: Mycobacterium tuberculosis MmsA (Rv0753c) Interacts with STING and Blunts the Type I Interferon Response
Source: mBio. 2020 Dec 1;11(6):e03254-19. doi: 10.1128/mBio.03254-19 (PMC7733952; doi:10.1128/mBio.03254-19)
Supplement: TABLE S3 [file mBio.03254-19-st003.docx]

**Table S3 List of primers for qPCR analysis.**

| **Target gene** | **Gene ID** | **Sequence** |
| --- | --- | --- |
| *Murine GAPDH* | 14433 | F: GAGCCAAACGGGTCATCATCT |
|  |  | R: GAGGGGCCATCCACAGTCTT |
| *Murine IFN-β* | 15977 | F: ctggagcagctgaatggaaag |
|  |  | R: cttgaagtccgccctgtaggt |
| *Murine STING* | 72512 | F: GGTCACCGCTCCAAATATGTAG |
|  |  | R: CAGTAGTCCAAGTTCGTGCGA |
| *Mtb SigA* | 887477 | F: TCGAGGTGATCAACAAGCTG |
|  |  | R: CTGCAGCAAAGTGAAGGACA |
| *Mtb mmsA* | 888707 | F: GGGAAGTTGAACGGGGTGAT |
|  |  | R: CATTCCCCACCTGCTCAAGG |
